# Genomic Analysis of Uterine Lavage Fluid Detects Early Endometrial Cancers and Reveals a Prevalent Landscape of Driver Mutations in Women without Histopathologic Evidence of Cancer: A Prospective Cross-Sectional Study

**DOI:** 10.1371/journal.pmed.1002206

**Published:** 2016-12-27

**Authors:** Navya Nair, Olga Camacho-Vanegas, Dmitry Rykunov, Matthew Dashkoff, Sandra Catalina Camacho, Cassie A. Schumacher, Jonathan C. Irish, Timothy T. Harkins, Elijah Freeman, Isaac Garcia, Elena Pereira, Sviatoslav Kendall, Rachel Belfer, Tamara Kalir, Robert Sebra, Boris Reva, Peter Dottino, John A. Martignetti

**Affiliations:** 1 Department of Obstetrics, Gynecology and Reproductive Sciences, Icahn School of Medicine at Mount Sinai, New York, New York, United States of America; 2 Department of Genetics and Genomic Sciences, Icahn School of Medicine at Mount Sinai, New York, New York, United States of America; 3 Swift Biosciences, Ann Arbor, Michigan, United States of America; 4 Jefferson School of Medicine, Philadelphia, Pennsylvania, United States of America; 5 Department of Pathology, Icahn School of Medicine at Mount Sinai, New York, New York, United States of America; 6 Department of Oncological Sciences, Icahn School of Medicine at Mount Sinai, New York, New York, United States of America; 7 Laboratory for Translational Research, Western Connecticut Health Network, Danbury, Connecticut, United States of America; Washington University School of Medicine, UNITED STATES

## Abstract

**Background:**

Endometrial cancer is the most common gynecologic malignancy, and its incidence and associated mortality are increasing. Despite the immediate need to detect these cancers at an earlier stage, there is no effective screening methodology or protocol for endometrial cancer. The comprehensive, genomics-based analysis of endometrial cancer by The Cancer Genome Atlas (TCGA) revealed many of the molecular defects that define this cancer. Based on these cancer genome results, and in a prospective study, we hypothesized that the use of ultra-deep, targeted gene sequencing could detect somatic mutations in uterine lavage fluid obtained from women undergoing hysteroscopy as a means of molecular screening and diagnosis.

**Methods and Findings:**

Uterine lavage and paired blood samples were collected and analyzed from 107 consecutive patients who were undergoing hysteroscopy and curettage for diagnostic evaluation from this single-institution study. The lavage fluid was separated into cellular and acellular fractions by centrifugation. Cellular and cell-free DNA (cfDNA) were isolated from each lavage. Two targeted next-generation sequencing (NGS) gene panels, one composed of 56 genes and the other of 12 genes, were used for ultra-deep sequencing. To rule out potential NGS-based errors, orthogonal mutation validation was performed using digital PCR and Sanger sequencing.

Seven patients were diagnosed with endometrial cancer based on classic histopathologic analysis. Six of these patients had stage IA cancer, and one of these cancers was only detectable as a microscopic focus within a polyp. All seven patients were found to have significant cancer-associated gene mutations in both cell pellet and cfDNA fractions. In the four patients in whom adequate tumor sample was available, all tumor mutations above a specific allele fraction were present in the uterine lavage DNA samples. Mutations originally only detected in lavage fluid fractions were later confirmed to be present in tumor but at allele fractions significantly less than 1%. Of the remaining 95 patients diagnosed with benign or non-cancer pathology, 44 had no significant cancer mutations detected. Intriguingly, 51 patients without histopathologic evidence of cancer had relatively high allele fraction (1.0%–30.4%), cancer-associated mutations. Participants with detected driver and potential driver mutations were significantly older (mean age mutated = 57.96, 95% confidence interval [CI]: 3.30–∞, mean age no mutations = 50.35; *p*-value = 0.002; Benjamini-Hochberg [BH] adjusted *p*-value = 0.015) and more likely to be post-menopausal (*p*-value = 0.004; BH-adjusted *p*-value = 0.015) than those without these mutations. No associations were detected between mutation status and race/ethnicity, body mass index, diabetes, parity, and smoking status. Long-term follow-up was not presently available in this prospective study for those women without histopathologic evidence of cancer.

**Conclusions:**

Using ultra-deep NGS, we identified somatic mutations in DNA extracted both from cell pellets and a never previously reported cfDNA fraction from the uterine lavage. Using our targeted sequencing approach, endometrial driver mutations were identified in all seven women who received a cancer diagnosis based on classic histopathology of tissue curettage obtained at the time of hysteroscopy. In addition, relatively high allele fraction driver mutations were identified in the lavage fluid of approximately half of the women without a cancer diagnosis. Increasing age and post-menopausal status were associated with the presence of these cancer-associated mutations, suggesting the prevalent existence of a premalignant landscape in women without clinical evidence of cancer. Given that a uterine lavage can be easily and quickly performed even outside of the operating room and in a physician’s office-based setting, our findings suggest the future possibility of this approach for screening women for the earliest stages of endometrial cancer. However, our findings suggest that further insight into development of cancer or its interruption are needed before translation to the clinic.

## Abstract 2

Navya Nair and colleagues describe a screening method for early identification of endometrial cancer. This method also reveals cancer mutations in women without cancer.

## Author Summary

**Why Was This Study Done?:**

- Endometrial cancer is the most common gynecologic malignancy in the United States.
- When detected early, endometrial cancer survival rates are improved.
- There are no screening methods that can detect either pre-malignant lesions or early-stage cancers.

**What Did the Researchers Do and Find?:**

- We conducted a prospective study in 107 women where uterine lavage fluid was analyzed for genetic mutations using ultra-deep, panel-based next-generation sequencing (NGS).
- Seven of the 107 women were identified by gold-standard histopathology as having endometrial cancer. All seven, even those with microscopic cancer foci, had significant cancer-associated gene mutations detected in their lavage fluid.
- Fifty-one women without histopathologic evidence of cancer had high allele fraction cancer-associated mutations.

**What Do These Findings Mean?:**

- This study provides the first evidence for the ability of an NGS-based approach to prospectively detect early-stage, microscopic endometrial cancer.
- Our study identified a previously unknown but highly prevalent landscape of driver and potential driver mutations in women who did not have histopathologic evidence of endometrial cancer.
- These findings suggest that while NGS-based analysis of uterine lavage can achieve the necessary sensitivity for endometrial cancer screening, further insights into the steps leading to endometrial cancer development and/or its interruption are still needed before this goal can be achieved.
- These findings, based on ultra-deep sequencing, may also have implications for understanding the development and clonal expansion of somatic cancer-driver mutations in apparently non-diseased tissues.

## Introduction

Endometrial cancer is the most common gynecologic malignancy in the United States, with 60,000 incident cases and greater than 10,000 deaths estimated for 2016. Alarmingly, both the incidence and associated mortality are rising [1]. By 2030, endometrial cancer is projected to surpass colorectal cancer to become the third most common cancer among women in the United States [2]. Despite its already high prevalence and increasing morbidity and mortality, no effective screening exists for endometrial cancer. Specifically, no screening methods can effectively detect either pre-malignant lesions (primary prevention) or early-stage cancers (secondary prevention). The lack of screening is particularly significant because when detected early, endometrial cancer survival rates are dramatically improved. The 5-year survival for localized disease is 95%, whereas it is <20% for disease that has metastasized [3].

Postmenopausal bleeding is the most common presenting symptom for women with endometrial cancer. Abnormal bleeding is reported in ~90% of cases [4] and simultaneously is one of the most common reasons for an office gynecology visit [5]. Conversely, and dependent upon risk factors, less than 10% of these women will have endometrial cancer [6,7]. Uterine fibroids, adenomyosis, polyps, and ovulatory dysfunction represent the most common causes of bleeding. Currently, the direct visual inspection of the uterine cavity through hysteroscopy combined with curettage of tissue or complete hysterectomy are considered “gold standards” for evaluating endometrial pathology and diagnosing endometrial cancer [8]. Both procedures entail the use of an operating room setting, patient anesthesia, some degree of patient discomfort, and high costs. The optimal screening test would avoid many of these issues while offering the ability to reliably detect all endometrial cancers at the earliest stage.

For more than 70 years, it has been appreciated that endometrial cancer and its precursors exfoliate cells into the uterine cavity [9], and, for almost as long, attempts have focused on obtaining these cancerous/precancerous cells for diagnostic purposes [10]. The success in marked mortality reduction in cervical cancer, another gynecologic cancer, through the use of the Papanicolaou (Pap) test provides the driving rationale for endometrial cancer screening. In this relatively simple test, a simple brushing or scraping of the cervix provides a sampling of cells for histologic evaluation of premalignant changes [11].

The first description of using uterine lavage for endometrial cancer detection was in 1957 [12]. Saline was introduced into the uterine cavity and then returned via aspiration. Cells within this lavage were centrifuged, smeared onto slides, and then evaluated by a cytopathologist [12]. A number of issues, including overall accuracy in cancer identification, difficulties in handling and processing of the aspirate, and requirement for cytopathology expertise, all limited the clinical adoption of this technique [10]. Nonetheless, a number of investigators have used variations upon this uterine lavage theme for attempts at developing screening and/or diagnostic tests. These include: collecting cells for cytology during ultrasound evaluation of the uterus [13,14], evaluating matrix metalloproteinase levels from women with endometrial cancer [15], and measuring DNA microsatellite instability in Lynch syndrome patients with endometrial cancer [16].

Recently, two exciting proof-of-principle studies demonstrated the use of next-generation sequencing (NGS) of DNA of uterine shed cells to identify somatic mutations in patients with known gynecologic cancers [17,18]. In already established endometrial and ovarian cancer cases, the authors demonstrated that panel-based, targeted sequencing of shed cells, retrieved either by brushing of the cervical canal [17] or through uterine lavage [18], could detect somatic mutations consistent with these two Müllerian duct-derived cancer types. In part, these two studies were made possible by the in-depth genetic characterization of endometrial cancers by The Cancer Genome Atlas Research Network (TCGA). TCGA-derived data facilitated the design of targeted sequencing panels for more sensitive mutation detection given the succinct nature of the panels. [19] In general, the classification of endometrial cancers by mutational characteristics is reproducible and potentially an improved method of classification over traditional pathological diagnosis, which is known to be subjective and prone to error [20,21].

In this study, we sought to provide the first prospective analysis of uterine lavage fluid from women taken at the time of their evaluation for a definitive tissue-based diagnosis to assess the use of targeted NGS for detecting endometrial carcinomas. The women in this study were primarily either experiencing abnormal uterine bleeding or had abnormal pelvic ultrasound findings and were being evaluated by hysteroscopy and curettage for a tissue diagnosis. We obtained both cellular DNA present in uterine lavage fluid and cell-free DNA (cfDNA), which itself has never previously been described from the uterine cavity. Using first a pan-cancer 56-gene panel and then a TCGA-guided 12-gene endometrial cancer panel, we detected somatic mutations in all women who were later diagnosed with stage IA endometrial cancer. In addition, we determined that half of the women in our study who did not have clinical evidence of cancer nonetheless possessed a significant landscape of driver mutations at relatively high allele fractions. Our findings therefore suggest the apparently opposing possibilities of a genomics-based approach for endometrial cancer screening and the discovery of prevalent driver mutations in clinically defined non-cancerous tissue. Ultimately, these results may lead to further insights into the steps distinguishing between endometrial cancer development and its interruption.

## Methods

The study was conducted from September 2015 to November 2016. Patient samples were collected during the months of September 2015 to April 2016, with DNA extraction being performed concurrently with sample collection. NGS, Sanger sequencing, and digital droplet PCR were performed on these samples and validation sets from February 2016 to October 2016. Data analysis was performed once all samples were sequenced and histopathology results confirmed.

### Patient Enrollment and Sample Collection

All uterine lavage, blood, and tumor samples were collected in accordance with the Institutional Review Board of the Icahn School of Medicine at Mount Sinai at the time of the diagnostic procedure (GCO# 10–1166). All clinical investigation was conducted according to the principles expressed in the Declaration of Helsinki. Written informed consent was obtained from each enrolled patient. All patients undergoing hysteroscopy and dilation and curettage at our institution were offered the opportunity to enroll in the study. A total of 111 patients were enrolled and 107 samples collected from September 2015 to April 2016. Four participants did not undergo the scheduled procedure due to difficulties unrelated to our study but which precluded the surgeon from performing the procedure. Final diagnoses were available after completion of the molecular analyses, and seven patients were diagnosed with endometrial cancer by histopathology. Tumor tissue was available in sufficient amounts for research-based analysis from four of these seven patients.

### Uterine Lavage

Uterine lavage specimens were collected in the operating room at the time of hysteroscopy. Hysteroscopy was performed under either general or laryngeal mask airway anesthesia as deemed appropriate by the anesthesiologist. After induction of anesthesia, patients were placed in dorsal lithotomy position. A vaginal surgical prep with iodine was performed. Next, a speculum was placed in the vagina and the cervix was visualized. A tenaculum was used to grasp the cervix. If stenotic, dilators were used to dilate the cervix. The hysteroscope was advanced into the cervix and subsequently into the uterine cavity aided with either saline or glycine inflow. Immediately upon entering the uterine cavity with the hysteroscope, the initial 20–30 mL of fluid was collected using a 40 mL specimen trap device (Medline Mucus Specimen Trap 40cc, No. DYND44140 Venture Respiratory Inc, Brooklyn, NY) attached to suction. Following this collection, the patient underwent the remainder of their procedure as per their surgeon’s discretion.

### Lavage Processing

All uterine lavage samples in the specimen trap device were placed on ice and taken to the laboratory and processed within 1 hour of collection. In the laboratory, the lavage specimens were transferred to 50 mL centrifuge tubes (No. C1061, Denville Scientific Inc, Holliston, MA) and centrifuged at ~3,200 g for 20 min at 4°C. The acellular supernatant was separated from the cell pellet using a pipette and recentrifuged for an additional 10 min to remove any remaining cellular material and debris. This fraction was then collected and stored at -80°C until final DNA extraction.

The cell pellet was washed with red blood cell lysis solution (5 Prime, No. 2301310, Gaithersburg, MD) by adding 1 mL of the solution to the cell pellet, resuspending by gentle pipetting, incubating at room temperature for 5 min, then centrifuging at 420 g in a table top centrifuge for 5 min. The RBC lysis supernatant was then discarded, leaving behind the cell pellet. This was repeated until the cell pellet was cleared of visible red cell contamination. The cell pellet was stored at -80°C until DNA isolation was performed.

### DNA Isolation

Cell-free DNA (cfDNA) was first concentrated from the acellular portion using a centrifugal filter (Amicon Ultra-15 30 kiloDalton Filter Units, EMD Millipore, No. UFC903096, Darmstadt, Germany) into smaller volumes ranging from 0.5 to 2 mL using the manufacturer’s protocol. The concentrated cfDNA was then extracted (Circulating Nucleic Acid Kit, Qiagen, Hilden, Germany) and eluted with 105 uL of AVE buffer.

The efficiency of the cfDNA extraction process was initially tested by spiking each acellular lavage sample with a known concentration of HindIII digested lambda DNA (Qiagen, Hilden, Germany) prior to concentration and cfDNA isolation. Quantitative PCR was then used to quantify the DNA fragments. Based on the calculated amount of spiked DNA, extraction efficiency was estimated to be between 46% and 87%.

Cellular DNA was extracted from the cell pellets (ArchivePure DNA Kit, 5 Prime, Gaithersburg, MD) with a modified protocol to account for low cell density. Briefly, total centrifugation times were increased for the two DNA precipitation/wash steps. The centrifugation times were increased to 10 min and 5 min each for the isopropanol and ethanol washes, respectively. The precipitated cellular DNA was eluted in 35 uL of AVE buffer.

Germline DNA was isolated from 10 ml blood samples collected from each patient at the time of their hysteroscopy (K3 EDTA tubes, Fischer Scientific, Pittsburgh, PA). Germline DNA was isolated (ArchivePure DNA Kit, 5 Prime, Gaithersburg, MD) according to the DNA purification protocol for whole blood, as per the manufacturer’s protocol.

The DNA concentrations of all fractions were determined by QuBit fluorometry (ThermoFischer Scientific, Waltham, MA).

### Library Preparation and Next Generation Sequencing

For each patient, a set of sample trios from germline PBMC DNA and DNA isolated from the lavage cellular and acellular fractions were sequenced to an average of 5,000X coverage using a targeted amplicon panel. DNA sample quantity and integrity were assessed with an ALU repeat qPCR assay (Swift Biosciences, Ann Arbor, MI), and 10 ng qPCR quantified DNA was used as input into the Accel-Amplicon Panel. To establish baseline performance for the different sample types, sample trios from nine patients were initially sequenced using the Accel-Amplicon 56G Oncology Panel (Swift Biosciences, Ann Arbor, MI). Using the TCGA dataset for endometrial tumors and their associated mutational profiles, a smaller custom endometrial tumor amplicon panel was developed to cover the 12 genes with the highest mutation frequencies. These 12 genes included PTEN, PIK3CA, TP53, CTNNB1, KRAS, FGFR2, FBXW7, RB1, ATM, APC, ARID1A, and PIK3R1. This 12-gene panel includes 102 amplicons with an average length of 138 bp to maintain sensitivity with short, acellular DNA. The genomic target regions were designed to cover both hotspot loci and contiguous full-coding exons, including the full exonic coverage of TP53 (See S1 Table for a complete list of genomic loci covered).

To confirm patient identity and preserve proper sample assignments for each trio, a spike-in of a germline SNP panel was included in the 102-amplicon endometrial tumor panel, requiring 4% of sequencing reads. This collection of high minor allele fraction SNP variants provided robust discrimination among samples [22]. The low concentration spike-in enabled a 200X sequencing depth of SNP targets for germline variant calling while the oncology targets were simultaneously sequenced to a 5,000X sequencing depth for somatic variant calling.

### Next Generation Sequencing

Resulting targeted NGS libraries were quantified using qPCR and sequenced on an Illumina MiSeq using v2 chemistry. For data analysis, amplicon primers were trimmed using Cutadapt [23] and trimmed reads were aligned to the GRCh37 build of human genome using BWA [24]. Somatic variant calling was performed using MuTect, Varscan, and Lofreq after following GATK Best Practices. A target of 5,000x coverage and 10 ng inputs enabled the lower limit of detection to be set to the 1% fraction. The average performance metrics for each sample was 91% on target and 97% coverage uniformity as defined by 20% of the mean.

### Sequencing Validation

To minimize the potential for NGS-based sequencing errors, our protocol selected 30% of NGS-identified variants for validation using an orthogonal methodology. We used either digital droplet PCR, for those NGS-identified variants with allele fractions <10%, or Sanger sequencing, for those variants with allele fractions ≥10%. Custom TaqMan Assays (see S2 Table) were designed using the Life Technologies web-based design tool (http://www.thermofisher.com/order/custom-genomic-products/tools/genotyping/). Assays contained VIC or FAM labeled probes, which probed for the wild-type and mutant variants, respectively. Specificity of each assay was first validated by quantitative PCR. Next, sensitivity and lower limits of detection were established by digital droplet PCR (RainDance Technologies, Billerica, MA), as we have previously described [25]. When no tumor DNA was available, positive controls were synthesized and used for these reactions as 300–500 bp gBlocks Gene Fragments (Integrated DNA Technologies, Coralville, IA).

To examine the possibility that sequence artifacts were introduced during lavage cfDNA concentration, isolation, and purification, sheared control genomic DNA from well-characterized single (NA12878, Coriell Institute, Camden, NJ) and multiplexed (HD701, Horizon Diagnostics, Cambridge, UK) cell line references were processed through all of the steps as lavage cfDNA starting from dilution into 15 ml of saline. These control sample replicates were then sequenced at coverage levels greater (range: 12,000–26,000x) than the lavage samples.

### Nomination of Driver, Potential Driver, and Passenger Mutations

To rank affected patients, mutations were classified into three groups: “drivers,” “potential drivers,” and “passengers,” or “mutations of unknown significance.” For mutation classification, we used TCGA-defined endometrial cancer mutation statistics [26], the inclusive TCGA mutation statistics provided by the CBIO Cancer Genomics portal [27], and the COSMIC database [28]; the functional impact of sequence variants was assessed by Mutation Assessor [29]. The group of driver mutations included those in activating or inactivating hotspots of oncogenes or tumor suppressors in major endometrial cancer driver genes of our panel, as well as truncating mutations in tumor suppressor genes of endometrial cancer. Thus, all mutations nominated as drivers were previously observed in endometrial cancer [26] and are also recurrent pan-cancer mutations [30]. In the group of “potential drivers,” we included predicted functional missense mutations in major endometrial cancer genes from the 12-gene panel. Mutation-drivers are typically observed in evolutionarily conserved positions and therefore assessed as functional by a Mutation Assessor score [29]. This result justifies nomination of driver mutation using a shorter list of predicted functional mutations, rather than the “long tail” of all mutations [31,32].

## Results

### Study Design and Patient Demographics

**Fig 1:**
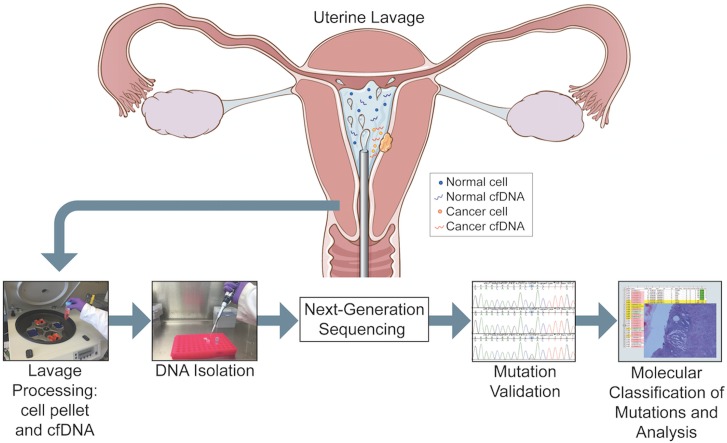
Overview of the study pipeline beginning with collection of uterine lavage fluid at the initiation of hysteroscopy. Figure designed by Jill Gregory.

An overview of the study pipeline is presented in Fig 1. The first 111 women who were scheduled to be evaluated by hysteroscopy and curettage for a tissue-based diagnosis were enrolled in the study. Endometrial lavage samples were collected from 107 patients (Table 1). The most frequent preoperative diagnoses were abnormal uterine bleeding (*n* = 50, 46.7%), uterine polyp (*n* = 30, 38.0%), and thickened endometrium (*n* = 10, 9.3%). The most common indications for hysteroscopy in the general population include abnormal bleeding and structural uterine abnormalities [5]. In our cohort, this was also the case with the majority of patients undergoing hysteroscopy for abnormal bleeding, followed by various suspected structural abnormalities, including polyps, thickened endometrium, and fibroids suspected through initial ultrasound evaluation. Patients ranged in age from 29 to 85 y, with an average age of 57.5 y. The majority of patients were white (*n* = 70, 66.0%), with BMIs >25 (*n* = 66, 61.7%), and were post-menopausal (*n* = 59, 57.3%), parous (one or more children; *n* = 59, 57.8%), and non-smokers (*n* = 85, 80.2%) (Table 1).

**Table 1:** Baseline characteristics and pre- and post-hysteroscopy diagnoses of the patient cohort.

Demographic criteria	Number of patients (percentage)
**Age (in years) (*n* = 107)**	
<40	16 (5.2)
40–49	26 (24.7)
50–59	25 (23.8)
60–69	23 (21.9)
>70	17 (16.2)
**Race/Ethnicity (*n =* 106)**	
White	70 (66.0)
African-American	14 (13.2)
Asian	12 (11.3)
Hispanic	5 (4.7)
Other	5 (4.7)
**Menopausal status (*n* = 103)**	
Postmenopausal	59 (57.3)
Premenopausal	44 (42.7)
**Parity (*n* = 102)**	
0	43 (42.1)
1–2	37 (36.3)
3–5	18 (17.7)
>5	4 (3.9)
**Smoking status (*n* = 106)**	
Never smoker	85 (80.2)
Current everyday smoker	2 (1.9)
Former smoker	19 (17.9)
**Preoperative diagnosis (*n* = 107)**	
Abnormal bleeding (including postmenopausal bleeding)	50 (46.7)
Uterine polyp	30 (28.0)
Thickened endometrium seen on ultrasound	10 (9.3)
Uterine fibroid	6 (5.6)
History of endometrial hyperplasia or cancer	3 (2.8)
Pelvic pain	1 (0.9)
Other	7 (6.5)
**Final pathological diagnosis *(n =* 107)**	
Polyp or polypoid fragment	60 (56.1)
Normal endometrium	17 (15.9)
Fibroid	13 (12.1)
Endometrial cancer	7 (6.5)
Polyp and fibroid	5 (4.7)
Endometrial hyperplasia	3 (2.8)
Other	2 (1.9)

### Seven Endometrial Cancers Are Diagnosed by Tissue-Based Histopathology

**Fig 2:**
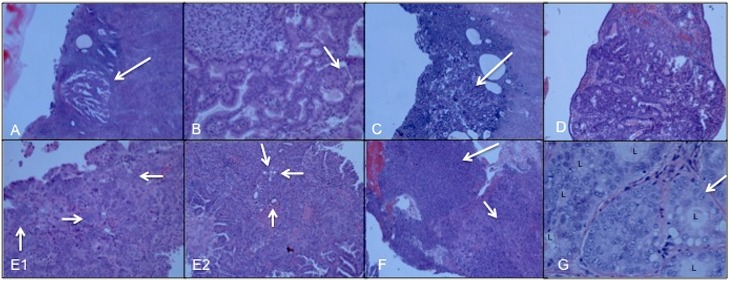
Microscopic views of hematoxylin-eosin stained sections of all seven uterine cancer specimens diagnosed by classic histopathology. (**A**) PT398: endometrioid, stage IA/grade 1 cancer. Arrow points to minute (<1 mm) focus of adenocarcinoma, adjacent to benign inactive endometrium (40x). (**B**) PT433: endometrioid, stage 1A/grade 1 cancer. Malignant glands organized in cribiform architecture. A small area of squamous differentiation is noted by the arrow (200x). (**C**) PT451: endometrioid, stage 1A/grade 1 cancer. Arrow points to a small focus of adenocarcinoma arising within the endometrial mucosa (40x). (**D**) PT484: endometrioid, stage 1A/grade 2 cancer arising within a polyp. Noted are cribiformed glands and complex architecture within the carcinoma (100x). (**E1**) PT468: fragment of high grade serous carcinoma with hyperchromatic nuclei (arrows) arranged in a complex glandular pattern (200x). (**E2**) PT468: another fragment of this tumor with grade 1–2 endometrioid carcinoma, with back-to-back glands (arrows) and lower-grade nuclei than the serous component presented in E1 (100x). (**F**) PT488: carcinosarcoma, stage 3A/grade 3 showing the biphasic features of high-grade carcinoma (top arrow pointing to the left of the image) and high-grade sarcoma (bottom arrow pointing to the right of the image) (40x). (**G**) PT492: endometrioid, stage IA/grade 1 cancer. High-power view of the cribiformed glands composed of malignant cells with atypical nuclei (arrow highlights one of these) depicting a grade 1 endometrioid adenocarcinoma ("L," lumen of the malignant glands) (400x).

All patients underwent uterine tissue curettage as part of the hysteroscopy, and the final diagnoses were determined by histopathologic assessment. The most frequent final diagnoses were polyp or polypoid fragment (*n* = 60, 56.1%), normal endometrium (*n* = 17, 15.9%), and fibroids (*n* = 13, 12.1%). Seven patients were diagnosed with cancer based on tissue analysis by histopathology (Table 2, Fig 2). A specialized gynecologic pathologist (T.K.) reviewed and verified each of these seven cases to confirm the diagnosis. Six of seven had stage IA cancer, and four of these were grade 1. One of these cancers was identified as a microscopic focus within a polyp and classified as <1 mm in size (Fig 2A). Four of seven were diagnosed with grade 1 endometrioid type cancer. The other three diagnoses were grade 2 endometrioid type, mixed grade 3 serous and grade 2 endometrioid type, and grade 3 carcinosarcoma. The clinicopathologic correlates of these cases are shown in Table 2.

**Table 2:** Clinicopathologic correlates of the seven cancer cases diagnosed by histopathology within the patient cohort.

Patient	Stage	Cancer histology and grade	Preoperative diagnosis	Age	Body Mass Index
PT398	IA	Endometrioid, G1	Endometrial polyp	72	21.3
PT433	IA	Endometrioid, G1	History of cancer, on progesterone	34	19.6
PT451	IA	Endometrioid, G1	Postmenopausal bleeding	65	45.0
PT468	IA	Mixed type: Serous, G3 & Endometrioid, G2	Postmenopausal bleeding	67	38.6
PT484	IA	Endometrioid, G2	Endometrial polyp	54	30.0
PT488	IIIA	Carcinosarcoma, G3	Postmenopausal bleeding	85	33.6
PT492	IA	Endometrioid, G1	Postmenopausal bleeding	82	26.1

### Identification of Somatic Mutations in Cellular and Acellular DNA Fractions from Lavage Fluid Collections

Lavage samples were collected from 107 patients and processed into cellular and acellular fractions following centrifugation. We had reasoned, based on previous studies, that lavage fluid should contain not only tumor cells [10,33,34] but also, given the origin of circulating free (cfDNA) and circulating tumor DNA (ctDNA) from apoptosing cells [35,36], cfDNA and ctDNA shed from the epithelial surface of the uterus containing normal, premalignant, and endometrial cancer cells. Endometrial cancers arise from cells in the inner lining of the uterus [37]. We therefore extracted DNA from the post-centrifuged cell pellet and acellular supernatant fractions. The average amount of DNA extracted from each cell pellet was 2,255 ng (range 1 ng–38,875 ng). In the acellular fraction, the average DNA amount was 2,046 ng (range 0.4 ng–44,572 ng). We quantified the extracted acellular DNA (2100 Expert Bioanalyzer, Agilent Technologies, Santa Clara, CA). A majority of the cfDNA fraction was approximately 175 bp in size (S1 Fig). This suggested not only size uniformity of the isolated DNA, inconsistent with contamination by randomly sheared genomic DNA arising from cells possibly within the collected lavage sample, but a size profile consistent with apoptotic fragmentation of genomic DNA at nucleosome ends.

**Fig 3:**
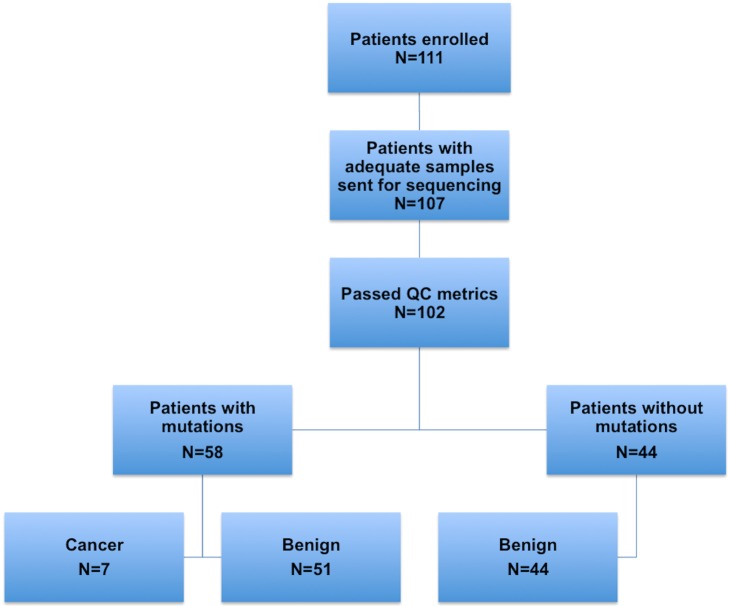
Flowchart depicting the numbers of patients in each step of the study: enrollment, sample collection, ultra-deep sequencing of samples, and molecular and histopathological classification.

**Fig 4:**
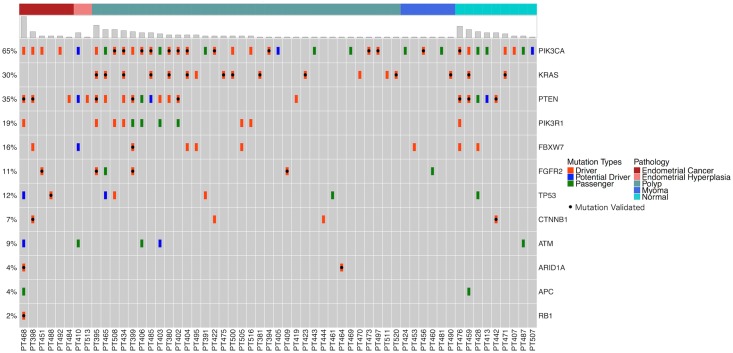
Mutation distributions detected by the 12-gene panel among the study cohort. Patients are represented along the *x*-axis first by their histopathologic diagnosis (represented in top bar) then by total mutation number. Mutation types were color-coded hierarchically, displaying the most consequential mutation type (driver, potential driver, passenger) detected at each patient/gene intersection, as some genes carried multiple mutations. NGS-defined mutations validated by dPCR or Sanger sequencing are represented by a black dot. Note: a number of genes had multiple mutations validated.

Samples from 102 patients passed all quality metrics, and only these were further analyzed. Nine patient samples were selected for sequencing using a targeted 56-gene, clinically relevant general oncology-related panel. In this pilot testing, four patients were found to have somatic mutations in their cellular and/or cfDNA. Based on these pilot results, we refined our sequencing strategy such that a targeted 12-gene endometrial cancer panel was used for all remaining samples. A flowchart describing the analysis and outcomes of the sequencing analysis is shown in Fig 3. In total, and based on results from both panels, 58 patients were found to have 126 unique somatic mutations in either the lavage cellular DNA or cfDNA (S3, S4 and S5 Tables). These included 89 missense, 24 nonsense (9 stop, 9 frame-shift, and 6 in-frame), and 13 silent mutations. Fifty-seven of these 58 patients had mutations detected by the 12-gene panel. A summary of the genes mutated, overall gene mutation frequencies, and correlation with histopathologic diagnosis is shown in Fig 4. Fourty-four patients had no mutations detected in either lavage cell pellet or cfDNA.

In total, 75 unique mutations were nominated as drivers (Table 3, S3 Table). Twenty-three mutations were nominated as “potential drivers,” 11 of which are recurrent mutations in that they are observed in other cancers, but not in endometrial cancer; two mutations were observed in endometrial cancer, but not in other cancers; and 10 mutations are newly described. The remaining 28 mutations were classified as “passenger” mutations or those having unknown significance.

**Table 3:** Cancer driver mutations identified in uterine lavage samples and their comparison to TCGA statistics.

Gene	Number of hotspot mutations in lavage samples (%)	Number of TCGA hotspots per gene (%)	Number of TCGA mutations per gene (% of samples affected)	Mutation frequency in hotspots**
PIK3CA	33 (32%)	86 (36%)	137 (57%)	H1047R (8/20); E545K/A (4/14); E542K/A (4/13); R88Q (2/11); Q546K (1/11); R93W/Q (3/6); M1043V (1/6); C420R (1/4); G106V (2/1); V344G(2/3); N345I/T(2/5); K111R(1/4); E453K(1/1); E81K(1/1)
KRAS	23 (23%)	45 (18%)	49 (20%)	G12D,V (21/36); G13C (1/9); Q61L (1/3)
PTEN	25 (25%)	62 (26%)	163 (67%)	R130G/Q/* (7/57); Y16* (1/3); A72fs (1/1); G132D (1/1); I33S (1/1); I67R (1/1); D92E (1/2); G165E (1/3); R173C (1/2); I32del; W111*; Y176del; L318fs; T321fs; F337fs; K342fs; P95L; K128Q; C211Y
PIK3R1	11 (11%)	3 (1%)	81 (34%)	D578H/A (2/5); T576delT (1/2); Y580fs (1/1); Y580D (1/3); R514C (1/1); R461* (1/2); **Y463_L466del; E558fs; L570fs; D464del**
ARID1A	5 (5%)	12 (5%)	82 (34%)	R1989* (1/9); R1722* (2/2); R1446* (1/1); E1444*
FGFR2	4 (4%)	9 (4%)	38 (16%)	S252W (4/9)
FBXW7	8 (8%)	6 (2%)	38 (16%)	R505C/G (3/6); R479Q (3/1); R465C (1/7); R441L (1/1)
TP53	2 (2%)	0	68 (21%)	R273H (1/9); S241F (1/3); **S166fs; Q165fs**
CTNNB1	6 (6%)	20 (8%)	73 (30%)	S37F (1/20); D32Y/A (2/12); S45F (2/3); T41A (1/5)
RB1	1 (1%)	0	25 (10%)	R445*

Total number of mutations detected in gene hotspots in this study and in endometrial tumors studied by TCGA [26]. The percentage of samples affected are given in parentheses.

**Numbers of mutations in hotspot positions detected, respectively, in lavage and in TCGA tumors are given in parentheses; novel mutations are highlighted in bold. Mutations that result in either a stop codon, in-frame deletion, or frame shift are marked, respectively, by “*,” “del,” and “fs” (e.g., W111*, Y463del, S116fs).

### Validation of NGS-Identified Mutations

As a validation assessment of the NGS-identified mutations, we selected a cohort of nearly one-third of all the mutations (55/184), across high and low allele fractions, for confirmation using two orthogonal, independent technologies. In total, 58 cellular DNA and/or cfDNA samples were thereby analyzed (S6 Table). For those mutations with allele fractions as defined by NGS as being ≥10%, we used Sanger sequencing; for allele fractions <10%, we used droplet digital PCR (ddPCR).

Notably, all mutations originally identified by NGS were validated. This included those variants with allele fractions as low as 1.0%, which was our lowest threshold for reporting variants by NGS. In addition to this analysis, when using ddPCR to validate a mutation identified in either the cellular DNA or cfDNA, the other paired specimen was also tested, even if no mutation was originally detected by NGS. In 14/15 sample pairings, the mutation was validated and confirmed to be present in the other sample and at an allele fraction of <1.0%; again, our original threshold cutoff. As shown in Table 4, the mutations identified in the lavage cell pellet and cfDNA were well correlated when binned by genes (*R*^2^ = 0.92, Pearson correlation coefficient) (Table 4; S2 Fig). Another interesting observation in those patients diagnosed with cancer by histopathology, as compared to all patients, is the increase in lavage-identified mutations with higher allele fractions (Table 4, bottom row). Specifically, for allele fractions of <5%, these fractions are 9% (cell pellet) and 15% (cfDNA) and for allele fractions of 5%–10%, the fractions are 27% (cell pellet) and 25% (cfDNA). Most notably, when the allele fractions are >10%, the fractions have increased to 50% (cell pellet) and 75% (cfDNA), and this is significant for both cfDNA (*p* = 0.0007, Fisher test) and cell pellets (*p* = 0.02), when the allele fraction is >10%.

**Table 4:** Summary of mutation allele fractions across patient samples by each mutated gene. Correlation between the total mutations/gene showed *R*^2^ = 0.92 (Pearson correlation coefficient) when compared between cell pellet DNA and cfDNA.

Gene	Cell pellet DNA			Sum of mutations	cfDNA			Sum of mutations	Mutation concordance		
	≤5.00%	5.01%–10.00%	≥10.01%		≤5.00%	5.01%–10.00%	≥10.01%				
	# mut	# mut	# mut		# mut	# mut	# mut		# mut	# unique patients	# cancer-diagnosed patients
**PTEN**	17	1	4	22	20	0	4	24	8	5	3
**KRAS**	16	1	2	19	10	1	1	12	7	6	0
**PIK3CA**	28	6	2	36	31	1	2	34	14	10	2
**TP53**	7	2	0	9	4	1	2	7	3	2	2
**PIK3R1**	7	1	0	8	10	0	0	10	3	2	0
**FGFR2**	3	2	0	5	3	0	0	3	2	2	1
**FBXW7**	5	1	1	7	4	0	0	4	1	1	0
**CTNNB1**	1	1	1	3	4	2	0	6	2	2	1
**ATM**	1	1	0	2	4	1	0	5	1	1	1
**APC**	1	0	0	1	2	0	0	2	1	1	1
**ARID1A**	3	0	1	4	2	0	2	4	3	1	1
**RB1**	1	0	1	2	0	0	2	2	2	1	1
**Total**	**90**	**16**	**12**	**-**	**94**	**6**	**13**	**-**	**47**		
# of cancer-diagnosed patients (total patients)	4 (43)	3 (11)	2 (4)	-	5 (33)	1 (4)	3 (4)	-	5 (19)	-	-

To rule out the possibility that sample preparation, occurring at any point starting from the actual uterine lavage collection, may have induced DNA artifacts, we attempted to replicate the same steps, including using the same data analysis pipeline, but using spiked-in control DNA samples. For this we chose two well-characterized sequencing reference controls. Genomic DNA isolated from the CEPH single cell line standard NA12878 and the multiplexed sample HD701, which represents a mixture of three different cell lines, was sheared (Covaris, Woburn, MA), size-selected, and then processed as all lavage cfDNA samples. NA12878 was processed in quadruplicate (i.e., the starting sample split into four and each sample processed and sequenced independently) and HD701 in duplicate. The sequencing coverage (range: 12,000–26,000x) was more than two-fold greater than that used for sequencing of the patient-derived lavage-isolated DNA samples. While all germline variants associated with these two control samples were identified at the appropriate allele fractions, no artifactual variants were identified across all replicates (S9 Table).

### Identification of Driver Mutations in Lavage Samples from All Cancer Cases

As noted above, seven of the patients in our cohort were diagnosed with clinical evidence of cancer following their hysteroscopy and tissue curettage (Table 2). All seven cases had somatic driver mutations identified in both the cell pellets and cfDNA isolated from their lavage fluid (S4 and S7 Tables). PT398 was diagnosed with stage 1A, grade 1 endometrioid endometrial adenocarcinoma, with a tumor measuring <1 mm in diameter contained within a polyp (Fig 2). Cellular DNA from the lavage fluid contained a total of six driver mutations, including three PTEN mutations (W111*, F337fs, G132D), one PIK3CA mutation (E545A), one CTNNB1 mutation (S45F), and one FBXW7 mutation (R505C). Two of these six driver mutations were also detected in the cfDNA. PT433, also diagnosed with stage 1A, grade 1 endometrioid endometrial adenocarcinoma, was one of the patients sequenced in the pilot study using the 56-gene panel. Six driver mutations were detected, five in RET (L773fs, E775_F776fs, F776L, V778fs, K780Q781fs) and one in CDH1 (K86fs). Another stage 1A grade 1 endometrioid endometrial adenocarcinoma patient, PT451, had two driver mutations detected in FGFR2 (S252W) and PIK3CA (M1043V) in both uterine lavage fractions. PT492, the fourth stage 1A, grade 1 endometrioid adenocarcinoma case, had one driver mutation detected in PIK3CA (G106V; cell pellet DNA). PT484, diagnosed with stage 1A, grade 2 endometrioid adenocarcinoma, had one driver mutation, PTEN (I67R), detected in both cellular DNA and cfDNA.

PT468 was diagnosed with a stage 1A mixed histology cancer, one component being high-grade serous adenocarcinoma and the other being grade 2 endometrioid adenocarcinoma. This patient had a total of ten driver mutations detected in the following four genes: ARID1A (R1722*, R1446*, R1989*), RB1 (R445*), PIK3R1 (R514C), PTEN (I33S, R130Q, C211Q), and PIK3CA (E81K, R88Q). With the exception of the ARID1A (R1722*), PIK3R1, and PTEN (C211Y) mutations, all other mutations were present in both cellular DNA and cfDNA fractions. In addition, there were six potential driver mutations detected in both or either one of the uterine lavage fractions. The diversity of mutations most likely reflects the diversity of the mixed histology tumor, distinguished by an aggressive high-grade serous component. PT488, the patient with stage 3A carcinosarcoma, had two TP53 mutations. One was classified as a driver (Q165fs) and the other a potential driver mutation (C176F). There was also an additional ARID1A driver mutation (E1444*). TP53 mutations have been shown to be present in aggressive endometrial adenocarcinomas, including high-grade serous types and carcinosarcomas [38]. ARID1A mutations have been shown to be associated with more aggressive endometrial adenocarcinomas [39].

Owing to the limited volume of three of these tumors (PT398, PT433, PT492), as per our IRB consent, there was no tumor tissue that could be made available for DNA isolation for research purposes. However, for four cases, we were able to isolate tumor DNA and compare paired tissue/lavage mutation profiles. DNA was extracted from fresh frozen tissue of these four tumor samples (PT451, PT468, PT484, PT488) and sequenced using the 12-gene panel. In all cases, at least one mutation present in the paired tumor DNA was detected in both the lavage cellular DNA and cfDNA (S7 Table). For some, but not all, of the tumor mutations, the allele fractions matched those from the lavage fractions. As noted in S7 Table, low allele fraction mutations in the tumor were not always detectable in the lavage fluid, and this varied by tumor.

Among these four cases in which the paired tumor was available, PT468 was unusual not only because of the large number of mutations identified (*n* = 20) but also because a large number of lavage-identified mutations had not been detected in the tumor (*n* = 14) and, conversely, one tumor-identified mutation was not detected in the lavage fluid (S7 Table). To determine the degree to which lavage-identified mutations could be present in the tumor, and again to investigate the possibility of artifact, we used ddPCR to interrogate the tumor DNA. We selected the eight mutations that were present in both cfDNA and cell pellet DNA (S8 Table) and designed probes for their analysis. There was a very high degree of concordance between the NGS- and ddPCR-defined allele fractions for all eight lavage mutations. Five of the eight mutations were confirmed to be present in the tumor (S8 Table, S3–S7 Figs), and these were present at allele fractions that would not have been detected by NGS, as our cutoff threshold for NGS was 1.0%. The allele fractions of these tumor variants ranged from 0.15% to 0.004%, whereas the cognate lavage fractions ranged from 2.0% to 21.0%.

### Identification of Frequent Driver Mutations in Women without Evidence of Cancer

In total, 95 women were diagnosed with benign or non-cancer conditions (Table 1). Sequencing of lavage fluid from 44 of these women identified no mutations. In marked contrast, 51 patients without a histopathologic diagnosis of cancer were identified as having somatic mutations in their uterine lavage samples. A total of 95 driver mutations were detected in this group, with 59 unique mutations.

The most frequent driver mutations detected among this group were KRAS G12C (eight patients), KRAS G12S (ten patients), and PIK3CA H1047R (eight patients) (S5 Table). The finding that a majority of women without a cancer diagnosis carried mutations, the relatively high allele fractions (range: 1.0% to 30.4%; average: 3.0%), and the projected oncogenic impact of these mutations was surprising. For example, PT395, with a histopathologic diagnosis of benign “polypoid fragments,” had 12 driver mutations detected in her uterine lavage. These affect a total of five genes: PTEN (I32del [AF: 2.1%], R130G [AF: 2.4%], G165E [AF: 2.3%]), PIK3R1 (Y463_L466del [AF: 5.5%], E558fs [AF: 1.6%]), PIK3CA (Q546K [AF: 5.1%], C420R [AF: 1.5%]), KRAS (G12S [AF: 1.2%], G12C [AF: 6.3%], G12C [AF: 1.4%]), and FGFR2 (S252W [AF: 5.8%]). Nine of these driver mutations were detected in cellular DNA, and five were also present in cfDNA. Two additional mutations were detected in the lavage cfDNA (KRAS 9G12S [AF: 1.2%], KRAS G12C [AF:6.3%]). To validate the existence of these mutations and exclude the possibility of sequencing artifacts, three of these 12 mutations were selected and tested by ddPCR (PTEN R130G, KRAS G12S, KRAS G12C; S5 and S6 Tables). All the mutations were confirmed. Another striking example is provided by PT485. She was diagnosed by tissue histopathology as having a benign polyp. We identified four driver mutations; three were detected in the cellular pellet ([KRAS G12S (AF: 1.1%)], PIK3CA [(H1047R (AF: 3.0%) and E542A (AF: 1.4%)]) and the fourth in the cfDNA (PIK3CA G106V; AF: 1.2%). Two of these four mutations (KRAS G12S and PIK3CA H1047R) were selected and validated by ddPCR (S6 Table).

**Fig 5:**
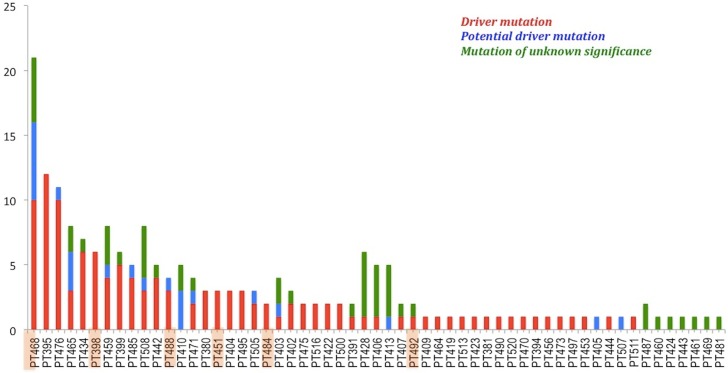
Patients with somatic mutations identified by NGS using the targeted 12-gene panel. Patients are ordered based on the sum of driver and potential driver mutations. Highlighted cases are those in which cancer was diagnosed by histopathology. Patient 433 is not represented because her samples were sequenced using the 56-gene panel.

A histogram of the mutation classifications, driver, potential driver, passenger, in all the patients is shown in Fig 5. The sum of driver and potential driver mutations, based upon the 12-gene panel, is plotted on the *y*-axis. The patients diagnosed by traditional histopathology cluster to the left of the graph.

To establish possible correlations between the presence of driver and/or potential driver mutations and clinical characteristics including age, race/ethnicity, BMI, diabetes, parity, smoking status, and menopausal status, we performed univariate analysis. Increasing age (*p*-value = 0.002; Benjamini-Hochberg [BH] adjusted *p*-value = 0.015; mean age no mutations = 50.35; mean age mutated = 57.96, 95% CI: 3.30–∞) and postmenopausal status (*p*-value = 0.004; BH-adjusted *p*-value = 0.015) were significantly associated with the presence of driver and potential driver mutations (S8 and S9 Figs). Increasing age was also found to be significantly associated with a diagnosis of cancer (*p*-value = 0.041) (S10 Fig).

We performed additional univariate analysis to further analyze our data and establish whether there is an association between mutations in each of the 12 genes on the targeted panel and the same clinical variables. Increasing age was significantly associated with presence of driver or potential driver mutations in PIK3CA (*p*-value = 0.008) and TP53 (*p*-value = 0.001) (S11 and S12 Figs).

## Discussion

This prospective study on women undergoing hysteroscopy and dilation and curettage as a diagnostic procedure demonstrated the ability of ultra-deep targeted sequencing of uterine lavage sampling to identify not only early-stage endometrial cancers but also a surprisingly high burden of driver mutations in a majority of women in our cohort who were without histopathologic evidence of cancer. As such, we believe these molecular findings simultaneously offer both the future promise of screening women for endometrial cancer while also providing a rare opportunity to explore the processes that are associated with the early steps of defining cancer development and its abrogation.

We specifically chose to examine all women, without any preselection for known genetic risk factors or family history of endometrial cancer, who were undergoing hysteroscopy for diagnostic evaluation. The most frequent reason for hysteroscopic evaluation in our cohort was abnormal uterine bleeding. Abnormal uterine bleeding is estimated to affect up 1.4 million women annually in the United States [40] and up to 14% of all women during their lifetime [41]. The most life-threatening etiology of this common gynecologic symptom is endometrial cancer. In women with abnormal uterine bleeding there is upwards of an ~10% risk of endometrial cancer, depending on age and menopausal status [42].Of the women in our cohort with a prediagnosis of abnormal uterine bleeding, 7% were found to have endometrial cancer and 49% were identified to be carrying driver/potential driver mutations but without a diagnosis of cancer.

The second most frequent reason for hysteroscopy in our cohort was the presence of an endometrial polyp. Endometrial polyps are associated with a small risk of cancer. In our cohort, Patient 484 was found to have a stage 1A, grade 2 endometrioid cancer arising from within a polyp. In general, upwards of 25% of women will be diagnosed with polyps at some point in their lives [43]. A number of studies have revealed that the overwhelming majority of polyps are clinically benign [43–45], 1.3%–6.0% are premalignant [45–48], 1.2%–3.9% display atypical hyperplasia [43,49,50], 10%–25.7% contain simple or complex endometrial hyperplasia [43,47,48], and 0.8%–3.5% are cancerous [43–45,51,52]. While polyps rarely become malignant, 10%–34% of cases of endometrial cancer in postmenopausal women are associated with polyps [53]. In our cohort, 34 women with polyps were not diagnosed with endometrial cancer by histopathology, while 31 possessed driver and/or potential driver mutations.

Endometrial hyperplasia or endometrial intraepithelial neoplasia is clinically relevant because it is a known precursor lesion to endometrial cancer [54]. An example of the associated risk for the development of cancer is provided by a study of 170 patients with all grades of endometrial hyperplasia, who did not undergo a hysterectomy for at least 1 y. Only 1% of patients with simple hyperplasia progressed to carcinoma, 2.0%–3.4% of patients with complex hyperplasia progressed to carcinoma, 10.5% of patients with complex hyperplasia progressed to atypical hyperplasia, and 23%–52% of atypical hyperplasia cases progressed to carcinoma [55,56]. The advancement from simple to complex and atypical hyperplasia takes years and is potentially influenced by factors such as specific genetic aberrations, patient age, BMI, and menopausal status [45]. Given the relative risks, it is therefore necessary to correctly distinguish between the grading categories, because these have direct relevance to cancer development risk and, thus, potential overtreatment or undertreatment. Based on our findings, we suggest that ultra-deep sequencing of lavage fluid is able to detect stage IA cancer and could also identify these premalignant lesions. Further studies will be necessary to test these hypotheses.

A decidedly unexpected outcome of these studies was the discovery that the majority of women without a cancer diagnosis in our cohort possessed somatic driver and candidate driver mutations within uterine lavage cells and cfDNA. Age (BH-adjusted *p*-value = 0.015) and postmenopausal status (BH-adjusted *p*-value = 0.015) were both positively associated with the likelihood of harboring these mutations. Therefore, from a clinical perspective, because of the prevalence of cancer-driver mutations identified in women without histopathologic evidence of cancer, our lavage screening protocol is not yet able to distinguish between women with and without clinically relevant evidence of cancer. However, our results seemingly provide a unique opportunity to gain insight into the mechanisms underlying selection and clonal expansion, as mutated cells evolve either towards a final cancer phenotype or are halted and eliminated in their progression. Based on the experience in our institution, it is expected that the overwhelming majority of women with a negative cancer diagnosis, based on the operating room procedure of combined hysteroscopy and tissue curettage, in the absence of continued symptoms will not develop clinically relevant endometrial cancer. The long-term surveillance of women in our study who possess these driver/candidate driver mutations will nonetheless provide clarification on the ultimate outcome.

Our assay was designed to have internal validation of NGS-based mutation calls using orthogonal detection methods, namely Sanger sequencing and digital PCR. Thus, the NGS-identified driver/candidate driver mutations in women without a diagnosis of cancer are not likely to represent technical artifacts because all variants that we tested by these two orthogonal technologies were validated. Nonetheless, because the possibility exists for “in vitro artifacts,” we do highlight this caveat and provide several arguments suggesting that, if they were present in our study, we do not believe they would undermine the main results. First, “in vitro artifacts” would be expected to affect both normal germline as well as “lavage” DNA. We did not observe any new, previously undocumented SNPs or deletions/insertions in germline controls. Second, while “in vitro artifacts” would be irrelevant to natural selection processes, the observed prevalence of detected mutations in common cancer hotspots (e.g., position G12 in KRAS) is more suggestive of an oncogenic selection process resulting in a typical cancer gene mutation distribution. Third, the comparison of the DNA mutation spectra in hotspot positions detected in this study with those reported by TCGA uterine cancer tumors is presented in S3 Table. The distribution of nucleotide mutations reveals that the most frequently observed TCGA mutations are (i) detected in our study and (ii) also detected as the most frequently occurring ones, e.g., mutation C>A, C>T in KRAS position 25398284; mutations A>G, G>A in PIK3CA positions 178952085 and 178936082, respectively; mutation C>G in PTEN position 89692904; and mutation G>C in FGFR2 position 123279677. Also, all hotspot mutations detected in our study are also reported in TCGA. The spectrum of TCGA mutations is, however, noticeably broader, which is not surprising given that the number of samples in their dataset was approximately ~3.5 times larger. In particular, percentages of DNA hotspot mutations reported in TCGA and not observed in our study are ~3% for KRAS; ~25% for PIK3CA; ~12% for PTEN; ~14% for FBXW7; and 33% for CTNBB1. For FGFR2 and ARID1A, we observed the same hotspot mutation types as reported in TCGA. Thus, our findings identify a clear tendency that the hotspot mutation spectra are basically similar for both studies. Finally, using spiked-in control DNA samples, we were not able to identify DNA artifacts introduced during the steps associated with lavage sample isolation and processing. Therefore, we believe it is unlikely that there is a strong in vitro artifact component affecting the observed spectrum of lavage mutations.

If "driver mutations" provide a selective growth advantage and can lead to cancer, how, then, does one explain the presence of high-frequency (allele fractions ranging from 1%–30%) driver/candidate driver mutations in half of our study population without cancer and who may have only a minimum risk of developing endometrial cancer. Three potentially instructive paradigms from recent genomic-based studies in other tissues from apparently healthy individuals may provide some insight. First, in normal blood and skin cells, driver mutations associated with clonal expansion have now been described [57]. Results from three nearly back-to-back whole-exome [58,59] and gene panel targeted sequencing studies [60] on nearly 34,000 individuals identified clonal hematopoiesis with leukemia-related, somatic driver mutations, most notably DNMT3A, in 10% of apparently healthy individuals >65 years of age and in nearly 20% in those between 90 to 108 years of age [58,59]. In individuals under <50 years of age, levels, while detectable, did not rise above 1%. While the absolute risk remained small, the individuals carrying these driver mutations were clearly at increased risk for developing future hematologic cancers, suggesting the premalignant nature of the detected clones [58,59].

Second, it has been appreciated for some time that clonal patches of skin contain TP53 mutations [61,62]. Recently, and using an ultra-deep sequencing strategy, it was shown that upwards of 32% of non-lesion-containing, sun-exposed epithelial cells from the eyelid contain mutations in key drivers of squamous cell carcinomas [63]. Finally, in a study searching for p53 mutations in peritoneal fluid, again using an ultra-deep sequencing strategy, all women in the study, 17 with ovarian cancer and 20 without evidence of cancer, were found to harbor TP53 mutations. For the women without cancer, the TP53 mutations were extremely low frequency (median mutant fraction <1/10,000) and associated with increasing age, but still were mostly deleterious and clustered in hotspots [64].

Taken together with our findings, genomic analysis is, thus, revealing a more complex genetic environment than previously believed to exist, in that some tissues, genetically-defined "driver mutations" are relatively frequent and prevalent in healthy individuals. Cancers do arise from clonal evolution and expansion of a single cell, and driver mutations confer a growth advantage to that cell [65,66]. But, as has been pointed out based on at least some of these findings [67], caution is needed prior to predicting clinical consequences or making patient-care decisions based solely upon gene mutations. Our NGS-based findings, and those by others [57–64], thus suggest that the evidence of potential malignancy may be determined well in advance of its pathologically defined appearance or clinical relevance. Given the limited size of the targeted panel used in our current study, it would seem reasonable to hypothesize that ultra-deep whole exome sequencing or whole genome sequencing would have identified an even higher percentage of women carrying cancer driver mutations but without a clinically defined cancer diagnosis. What may be most clinically relevant is identifying and understanding the mechanisms by which some clones continue to evolve and become cancer while others are halted. While successful screening for earlier detection of cancer will undoubtedly improve quality of life and improve survival, insights into halting the progression of steps linking somatic mutation and the evolution towards cancer provide the greatest benefits. These goals must be balanced by the harm that would currently be imposed by over-diagnosis.

In our study, we identified driver mutations in all seven women who were found to harbor endometrial cancers. Six of these were stage IA, the earliest cancer stage wherein the cancer either does not invade or does not invade beyond half of the myometrial tissue. Indeed, one of the stage IA cancers detected in our cohort was microscopic (Fig 2A), and for three others not enough tissue was available for additional research purposes. In detecting driver mutations in women with stage IA cancer, even in those cases in which only microscopic amounts of tissue were available and, thus, were a clinical challenge, we establish that adequate amounts of tumor shed cells and DNA are present in uterine lavage. Thus, at this time, ultra-deep sequencing represents a candidate screening methodology. Currently, there is no effective recommended screening method for endometrial cancer in the general population. Obtaining adequate endometrial tissue to establish a diagnosis is critically important. Endometrial sampling devices used in an office setting are currently the first line of evaluation. These methods are not always successful, particularly in postmenopausal women—exactly the population of women who are at highest risk of endometrial cancer. In these cases, hysteroscopy and curettage are used. Thus, novel methods are needed. Based on the results of this study, we propose that future clinical studies should address the possibility of uterine lavage as a potential screening test in women to detect endometrial cancers. Paradoxically, the same ability to detect mutations in women with endometrial cancer using ultra-deep sequencing, which promises the ability to screen for this cancer, also reveals a previously unknown prevalent landscape of driver mutations in women without clinically apparent evidence of cancer. This duality represents a diagnostic dilemma but an intriguing view towards new biologic questions. With the use of innovative technologies on the horizon, such as single-cell sequencing and further developments in NGS, we hope that these results will provide a catalyst for novel insights into endometrial cancer development and the protective mechanisms that limit the evolution of normal cells into cancer.

## Supporting Information

S1 Fig Representative size distribution of DNA extracted from the acellular lavage fraction. One of the major size peaks centers at approximately 175 bp.
(TIFF)

S2 Fig Correlation between the mutations identified in the lavage cell pellet and cfDNA when binned by genes (*R*^2^ = 0.92, Pearson correlation coefficient). (TIFF)

S3 Fig Detection of lavage-identified PIK3CA Chr3:178916876 G/A mutation in PT468 tumor DNA by digital droplet PCR assay. Top lettered panels correspond to the named samples in the table. Positive control: tumor DNA from an unrelated patient containing the same PIK3CA mutation. Negative controls: 66 ng of gDNA isolated from the patient’s PBMC. Tumor: 66 ng of tumor gDNA. Blank: no gDNA in PCR mix. VIC = wild-type allele, FAM = mutant allele.
(TIFF)

S4 Fig Detection of lavage-identified ARID1A Chr1:27105553 C/T mutation in PT468 tumor DNA by digital droplet PCR assay. Top lettered panels correspond to the named samples in the table. Positive control: synthesized gBlocks Gene Fragments (IDT) containing the specific mutation. Negative controls: 66 ng of gDNA isolated from the patient’s PBMC. Tumor: 66 ng of tumor gDNA. Blank: no gDNA in PCR mix. VIC = wild-type allele, FAM = mutant allele.
(TIFF)

S5 Fig Detection of lavage-identified ATM Chr11:108225584 C/A mutation in PT468 tumor DNA by digital droplet PCR assay. Top lettered panels correspond to the named samples in the table. Positive control: synthesized gBlocks Gene Fragments (IDT) containing the specific mutation. Negative controls: 66 ng of gDNA isolated from the patient’s PBMC. Tumor: 66 ng of tumor gDNA. Blank: no gDNA in PCR mix. VIC = wild-type allele, FAM = mutant allele.
(TIFF)

S6 Fig Detection of lavage-identified RB1 Chr13:48941662 T/G mutation in PT468 tumor DNA by digital droplet PCR assay. Top lettered panels correspond to the named samples in the table. Positive control: synthesized gBlocks Gene Fragments (IDT) containing the specific mutation. Negative controls: 66 ng of gDNA isolated from the patient’s PBMC. Tumor: 66 ng of tumor gDNA. Blank: no gDNA in PCR mix. VIC = wild-type allele, FAM = mutant allele.
(TIFF)

S7 Fig Detection of lavage-identified APC Chr5:112175664 C/A mutation in PT468 tumor DNA by digital droplet PCR assay. Top lettered panels correspond to the named samples in the table. Positive control: synthesized gBlocks Gene Fragments (IDT) containing the specific mutation. Negative controls: 66 ng of gDNA isolated from the patient’s PBMC. Tumor: 66 ng of tumor gDNA. Blank: no gDNA in PCR mix. VIC = wild-type allele, FAM = mutant allele.
(TIFF)

S8 Fig Comparison of patients with driver or potential driver mutations (50 patients, red histogram) with those that have only passenger or no mutations (52 patients, blue histogram) by age using one-tailed Mann-Whitney-Wilcoxon test. The distributions are statistically different: original *p*-value = 0.002, after BH multiple testing adjustment *p*-value = 0.015; the mean age for “No mutation” group is 50.35 y. The mean age for “Mutated” group is 57.96 y; difference = 7.61, 95% confidence interval for the difference (CI95%) = [3.30–∞].
(TIFF)

S9 Fig Comparison of patients with driver or potential driver mutations (45 patients, red histogram) with those that have only passenger mutations or no mutations (55 patients, blue histogram) by menopausal status using one-tailed Mann-Whitney-Wilcoxon test: original *p*-value = 0.003, BH-adjusted *p*-value = 0.015. (TIFF)

S10 Fig Comparison of patients with a cancer diagnosis by histopathology (seven patients, red histogram) and all other patients (95 patients, blue histogram) by age using one-tailed Mann-Whitney-Wilcoxon test: *p*-value = 0.021, BH-adjusted *p*-value = 0.08. The mean age “No cancer” is equal to 53.23 y, the mean age for “Cancer” group is equal to 65.57 y; difference = 12.34, CI95% = [-0.54–∞].
(TIFF)

S11 Fig Comparison of patients with PIK3CA mutations (37 patients, red histogram) and those without PIK3CA mutations (65 patients, blue histogram) by age using the one-tailed Mann-Whitney-Wilcoxon test: *p*-value = 0.004, BH-adjusted *p*-value = 0.033, the mean age for “No PIK3CA mutations” group is equal to 51.42 y, the mean age for “PIK3CA mutated” group = 58.76 y, difference = 7.34, CI95% = [2.77–∞]. (TIFF)

S12 Fig Comparison of patients with TP53 mutations (five patients, red histogram) and those without TP53 mutations (97 patients, blue histogram) by age using the one-tailed Mann-Whitney-Wilcoxon test: *p*-value = 0.001, BH-adjusted *p*-value = 0.005, the mean age for “No TP53 mutations” is equal to 52.98 y, the mean age for “TP53 mutated” group is equal to 75.40 y, difference = 22.42, CI95% = [15.57–∞]. (TIFF)

S1 Table Complete list of genomic loci covered in each gene, including hotspot loci and contiguous full coding exons, including the full exonic coverage of TP53 in the 12-gene panel. (XLSX)

S2 Table Taqman SNP assays used for validation of mutations identified by next generation sequencing. (XLSX)

S3 Table All lavage-identified cancer driver (3A), potential driver (3B), and passenger mutations (3C). (XLSX)

S4 Table All patients with somatic mutations identified by NGS of lavage fluid using the targeted 56-gene panel. "^," patients diagnosed with cancer by histopathology. "-," did not pass QC metrics.
(XLSX)

S5 Table All patients with somatic mutations identified by NGS of lavage fluid using the targeted 12-gene panel. "^," patients diagnosed with cancer by histopathology. "-," did not pass QC metrics. "NT," not tested.
(XLSX)

S6 Table List of mutations validated by ddPCR or Sanger sequencing. "^," patients with cancer as diagnosed by histopathology. "-," did not pass QC metrics. "NT," not tested.
(XLSX)

S7 Table Comparison of paired tumor and lavage mutations from four cancer cases. (XLSX)

S8 Table List of selected PT468 mutations validated by digital droplet PCR or Sanger sequencing. (XLSX)

S9 Table (A) All concordant variants identified by both GATK and LoFreq in the control sheared DNA sample replicates. All the variants identified, in both HD701 and NA12878, represent previously known and validated mutations from these genomic DNA sources. Note that in HD-701 replicate 1, the known ATM variant (chr11:108138045 C>T) detected in replicate 2 was only reported by LoFreq (allele fraction 6%, coverage 12,350, strand balance 0.059) and not by GATK and therefore variant not called. (B) Summary metrics for sequencing of the control sheared DNA sample replicates.
(XLSX)

S1 Text STROBE Checklist. (DOC)

## Abbreviations

cfDNA: cell-free DNA
ctDNA: circulating tumor DNA
ddPCR: droplet digital PCR
NGS: next-generation sequencing
Pap: Papanicolaou
TCGA: The Cancer Genome Atlas
