# Supplementary figures and images for: Genomic Analysis of Uterine Lavage Fluid Detects Early Endometrial Cancers and Reveals a Prevalent Landscape of Driver Mutations in Women without Histopathologic Evidence of Cancer: A Prospective Cross-Sectional Study
Source: PLoS Med. 2016 Dec 27;13(12):e1002206. doi: 10.1371/journal.pmed.1002206 (PMC5189938; doi:10.1371/journal.pmed.1002206)

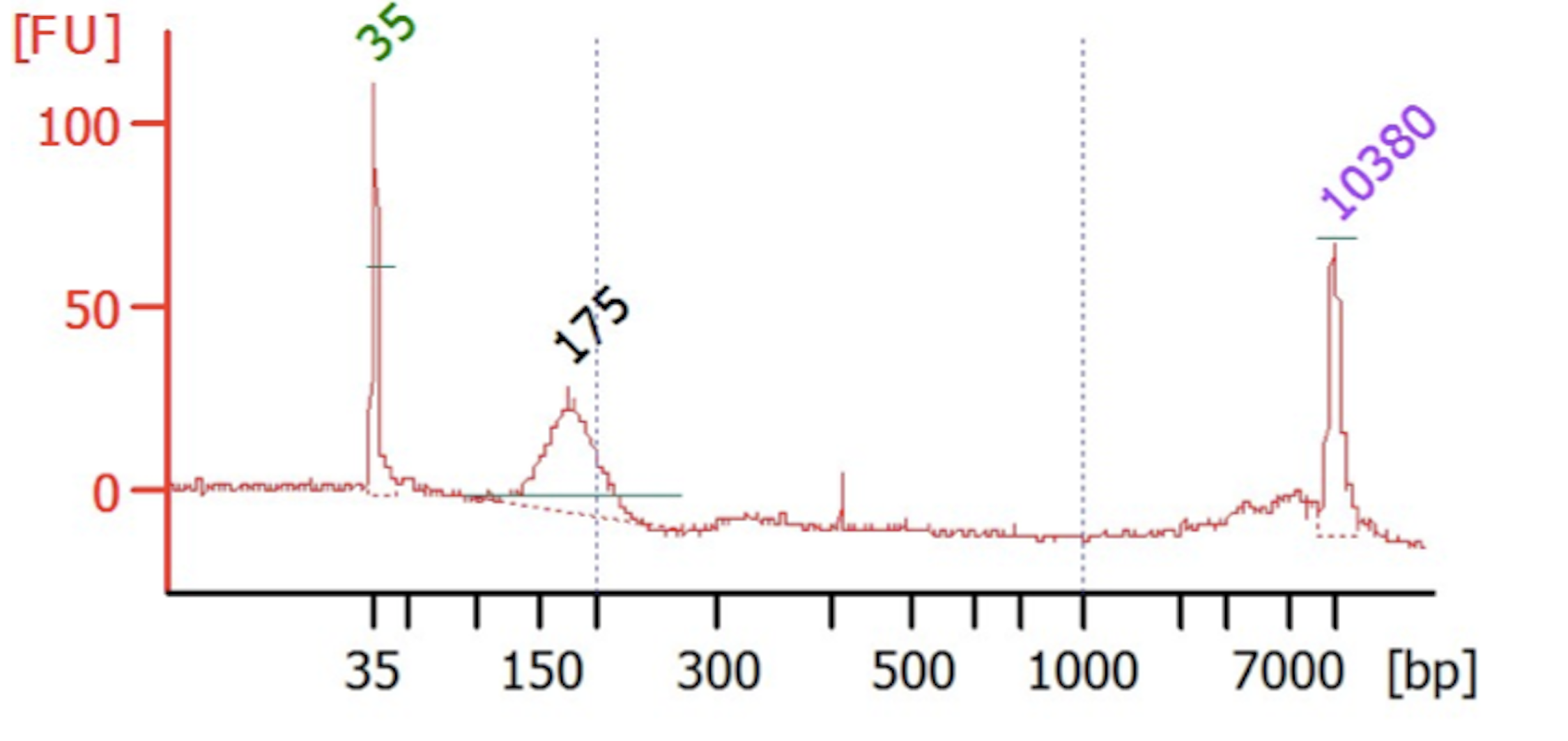

Supplement: S1 Fig — One of the major size peaks centers at approximately 175 bp. (TIFF) [file pmed.1002206.s001.tiff]

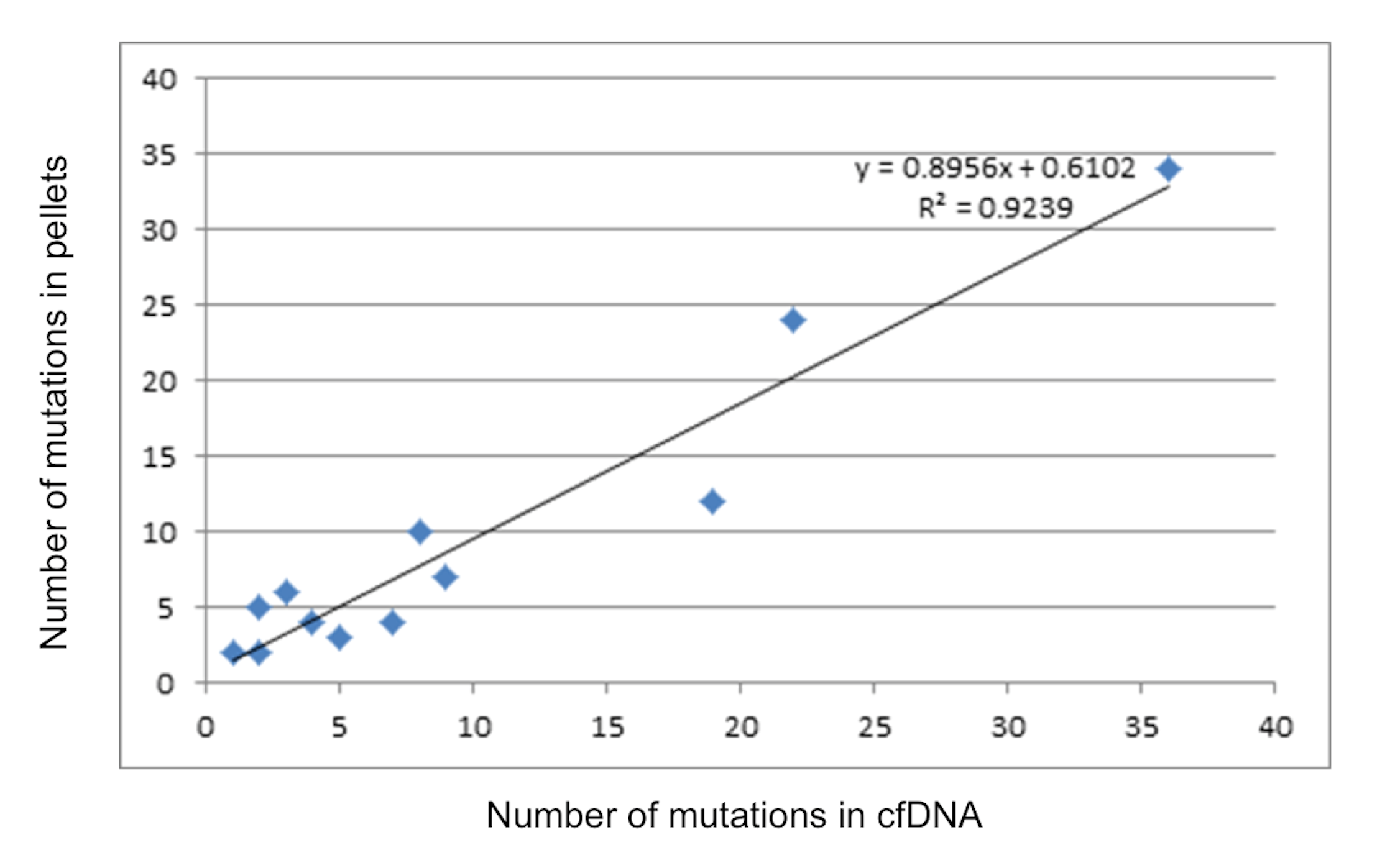

Supplement: S2 Fig — (TIFF) [file pmed.1002206.s002.tiff]

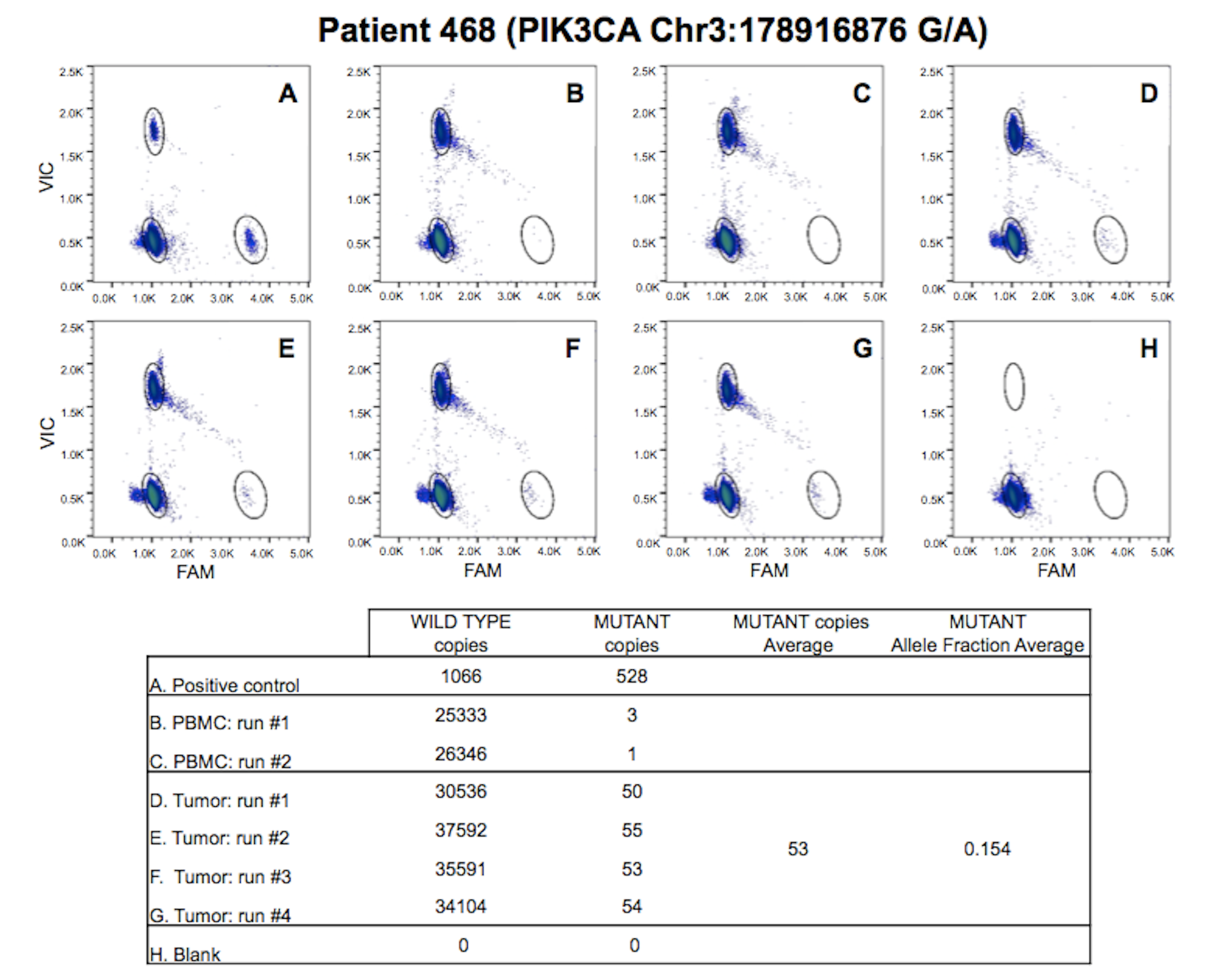

Supplement: S3 Fig — Top lettered panels correspond to the named samples in the table. Positive control: tumor DNA from an unrelated patient containing the same PIK3CA mutation. Negative controls: 66 ng of gDNA isolated from the patient’s PBMC. Tumor: 66 ng of tumor gDNA. Blank: no gDNA in PCR mix. VIC = wild-type allele, FAM = mutant allele. (TIFF) [file pmed.1002206.s003.tiff]

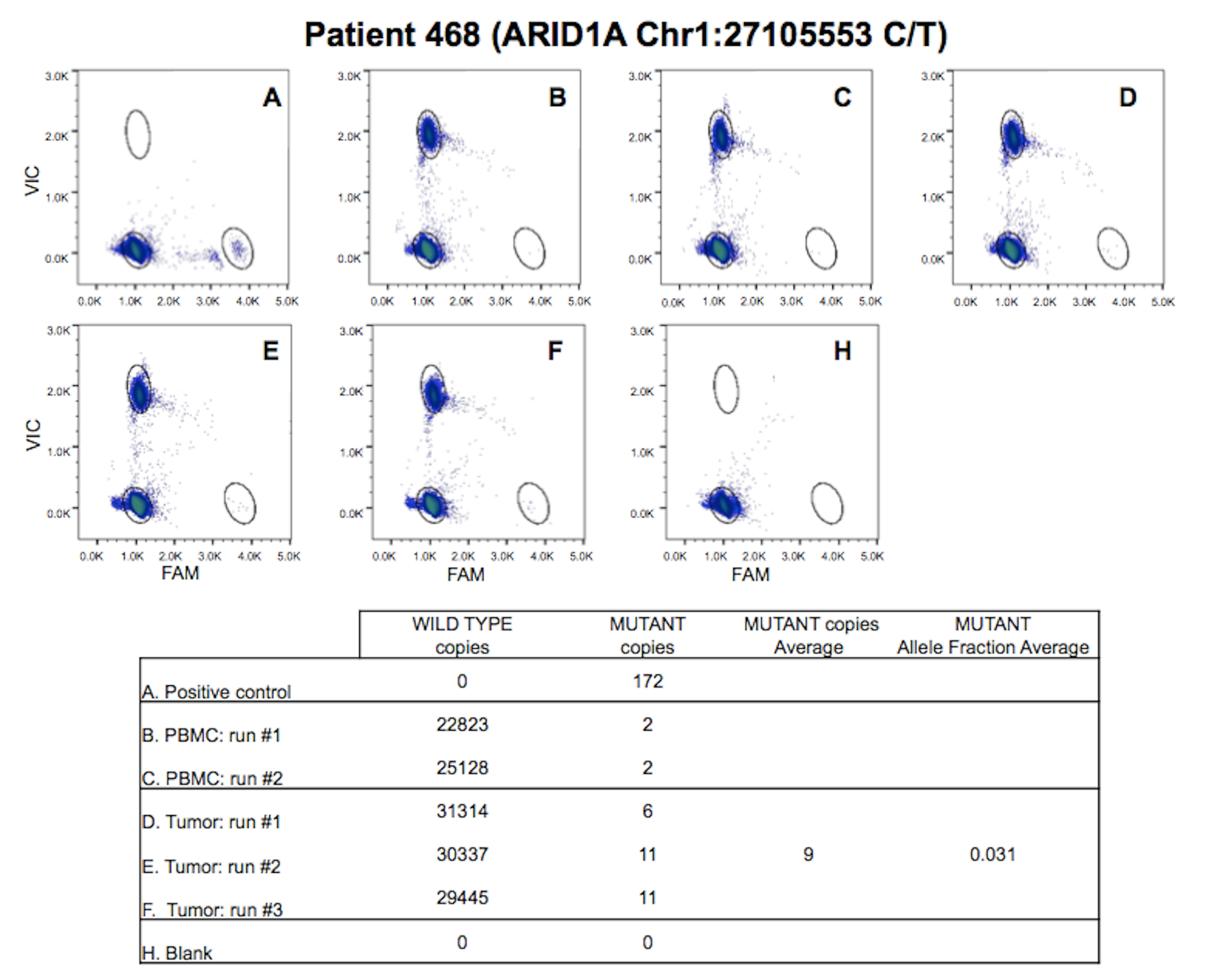

Supplement: S4 Fig — Top lettered panels correspond to the named samples in the table. Positive control: synthesized gBlocks Gene Fragments (IDT) containing the specific mutation. Negative controls: 66 ng of gDNA isolated from the patient’s PBMC. Tumor: 66 ng of tumor gDNA. Blank: no gDNA in PCR mix. VIC = wild-type allele, FAM = mutant allele. (TIFF) [file pmed.1002206.s004.tiff]

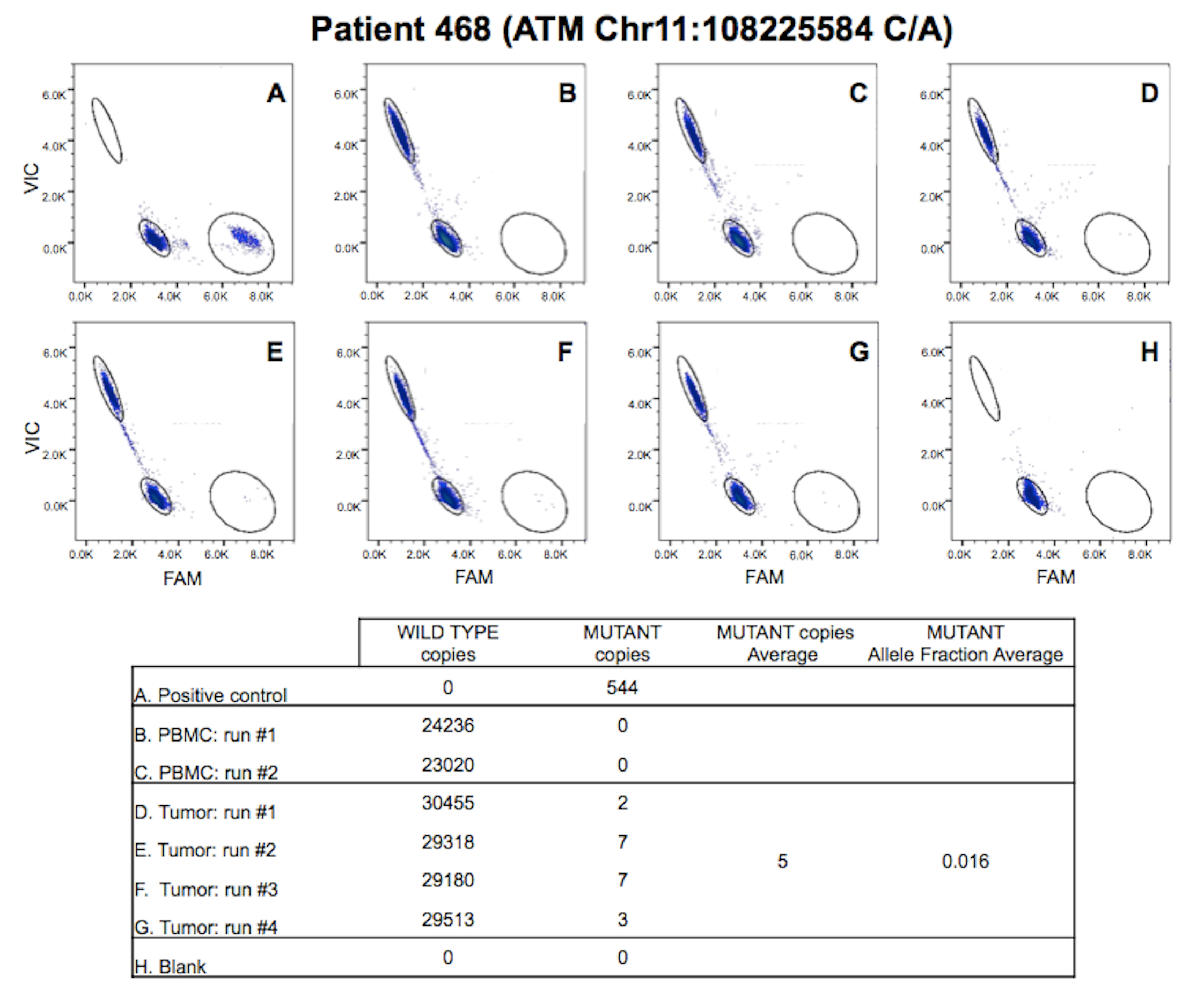

Supplement: S5 Fig — Top lettered panels correspond to the named samples in the table. Positive control: synthesized gBlocks Gene Fragments (IDT) containing the specific mutation. Negative controls: 66 ng of gDNA isolated from the patient’s PBMC. Tumor: 66 ng of tumor gDNA. Blank: no gDNA in PCR mix. VIC = wild-type allele, FAM = mutant allele. (TIFF) [file pmed.1002206.s005.tiff]

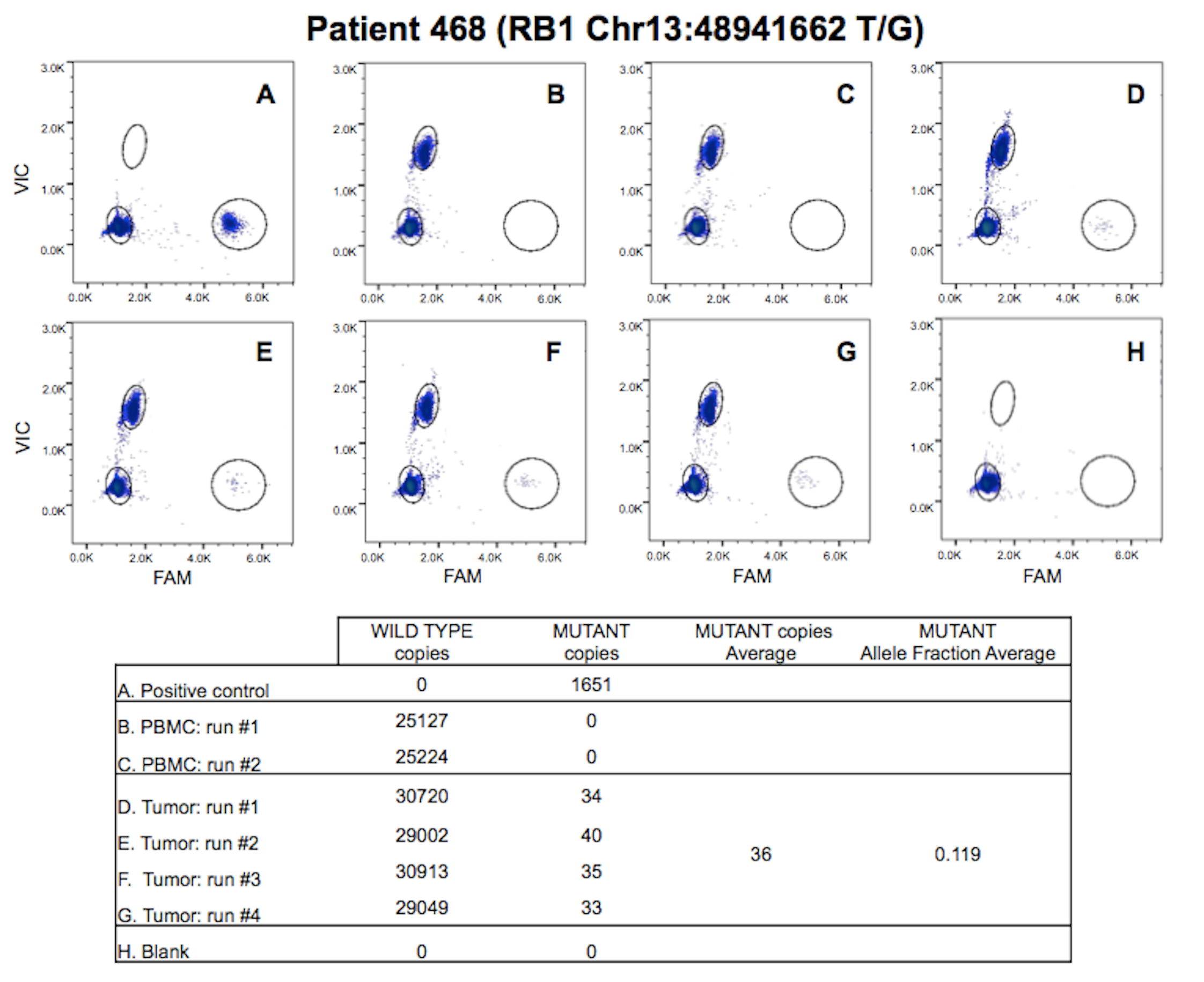

Supplement: S6 Fig — Top lettered panels correspond to the named samples in the table. Positive control: synthesized gBlocks Gene Fragments (IDT) containing the specific mutation. Negative controls: 66 ng of gDNA isolated from the patient’s PBMC. Tumor: 66 ng of tumor gDNA. Blank: no gDNA in PCR mix. VIC = wild-type allele, FAM = mutant allele. (TIFF) [file pmed.1002206.s006.tiff]

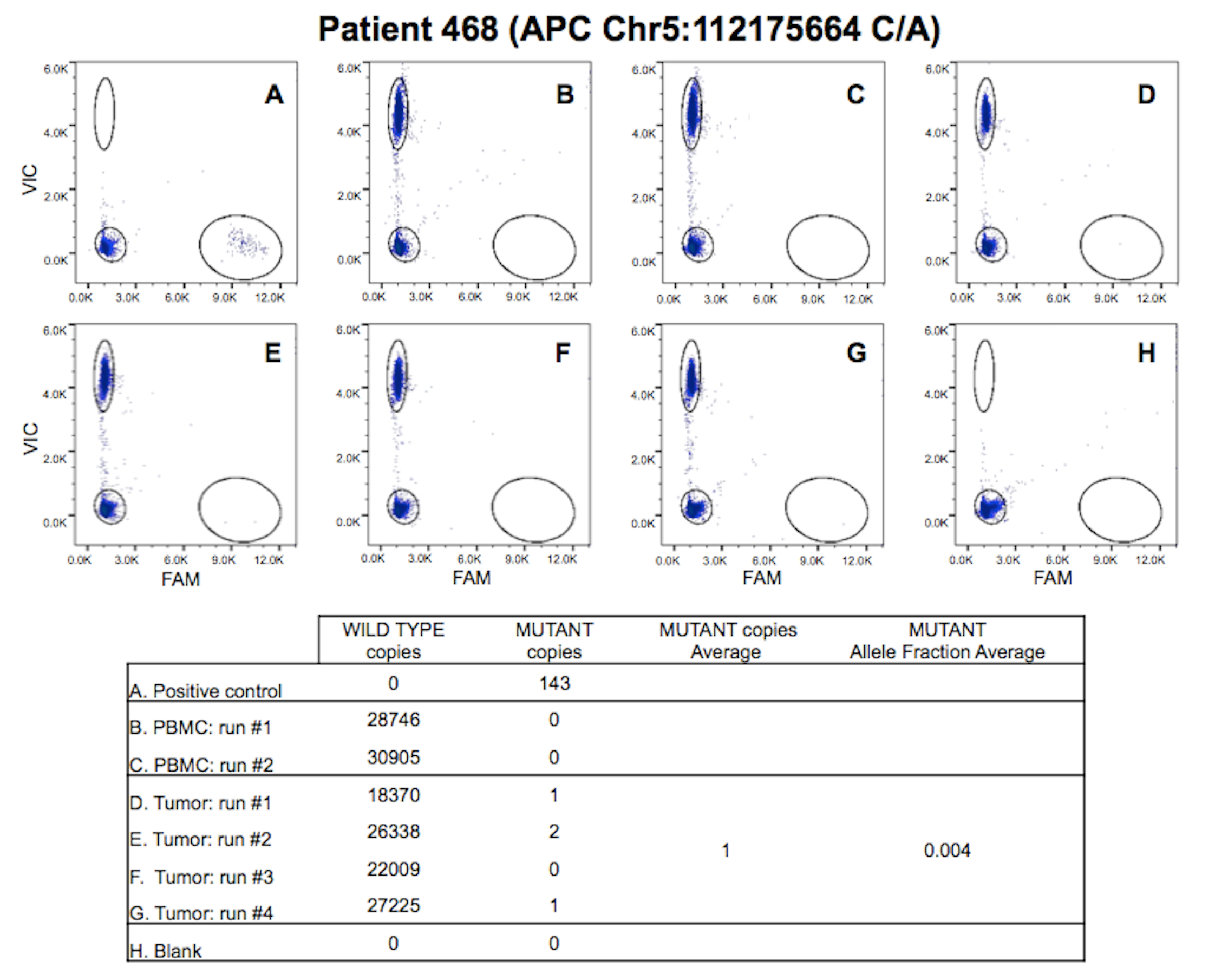

Supplement: S7 Fig — Top lettered panels correspond to the named samples in the table. Positive control: synthesized gBlocks Gene Fragments (IDT) containing the specific mutation. Negative controls: 66 ng of gDNA isolated from the patient’s PBMC. Tumor: 66 ng of tumor gDNA. Blank: no gDNA in PCR mix. VIC = wild-type allele, FAM = mutant allele. (TIFF) [file pmed.1002206.s007.tiff]

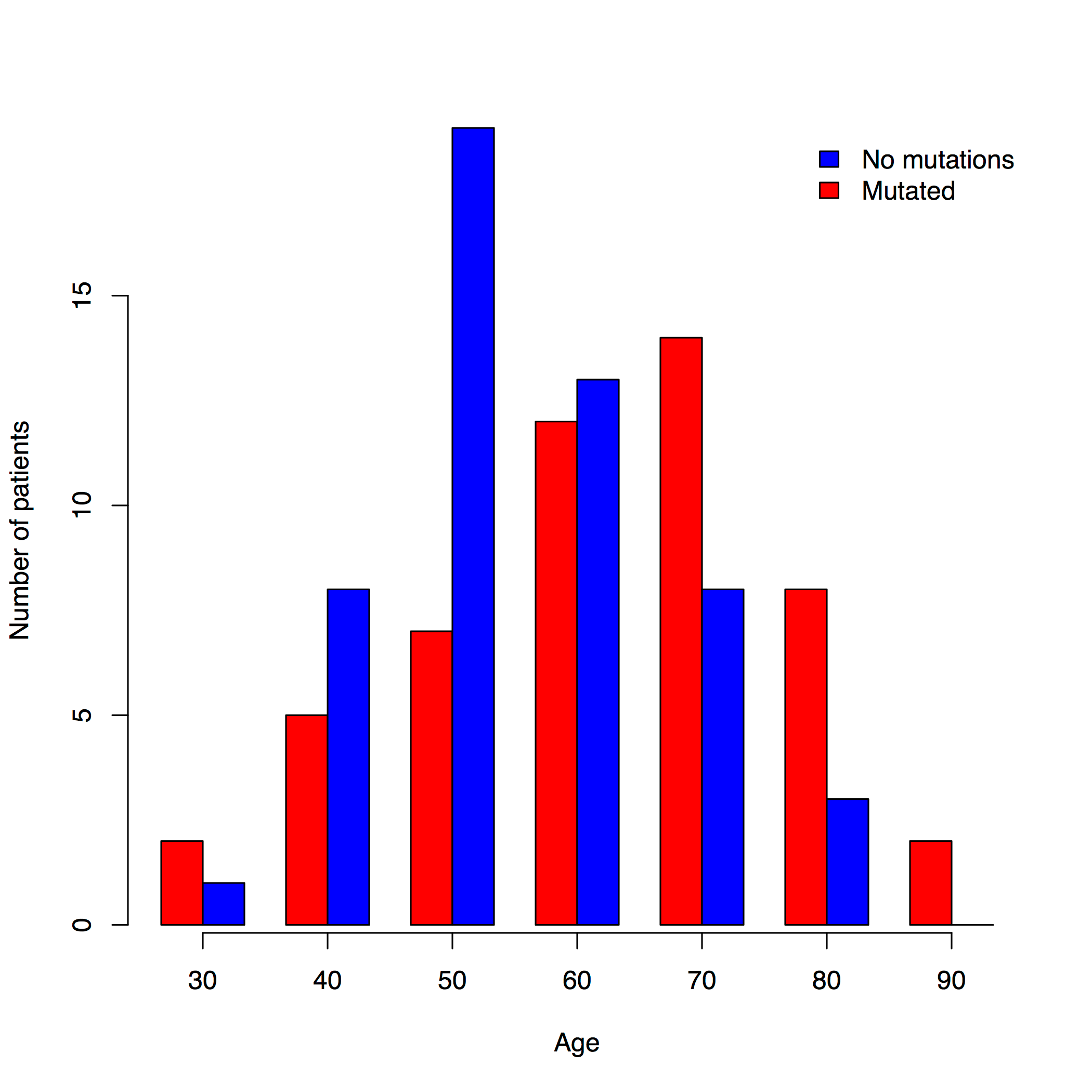

Supplement: S8 Fig — The distributions are statistically different: original p-value = 0.002, after BH multiple testing adjustment p-value = 0.015; the mean age for “No mutation” group is 50.35 y. The mean age for “Mutated” group is 57.96 y; difference = 7.61, 95% confidence interval for the difference (CI95%) = [3.30–∞]. (TIFF) [file pmed.1002206.s008.tiff]

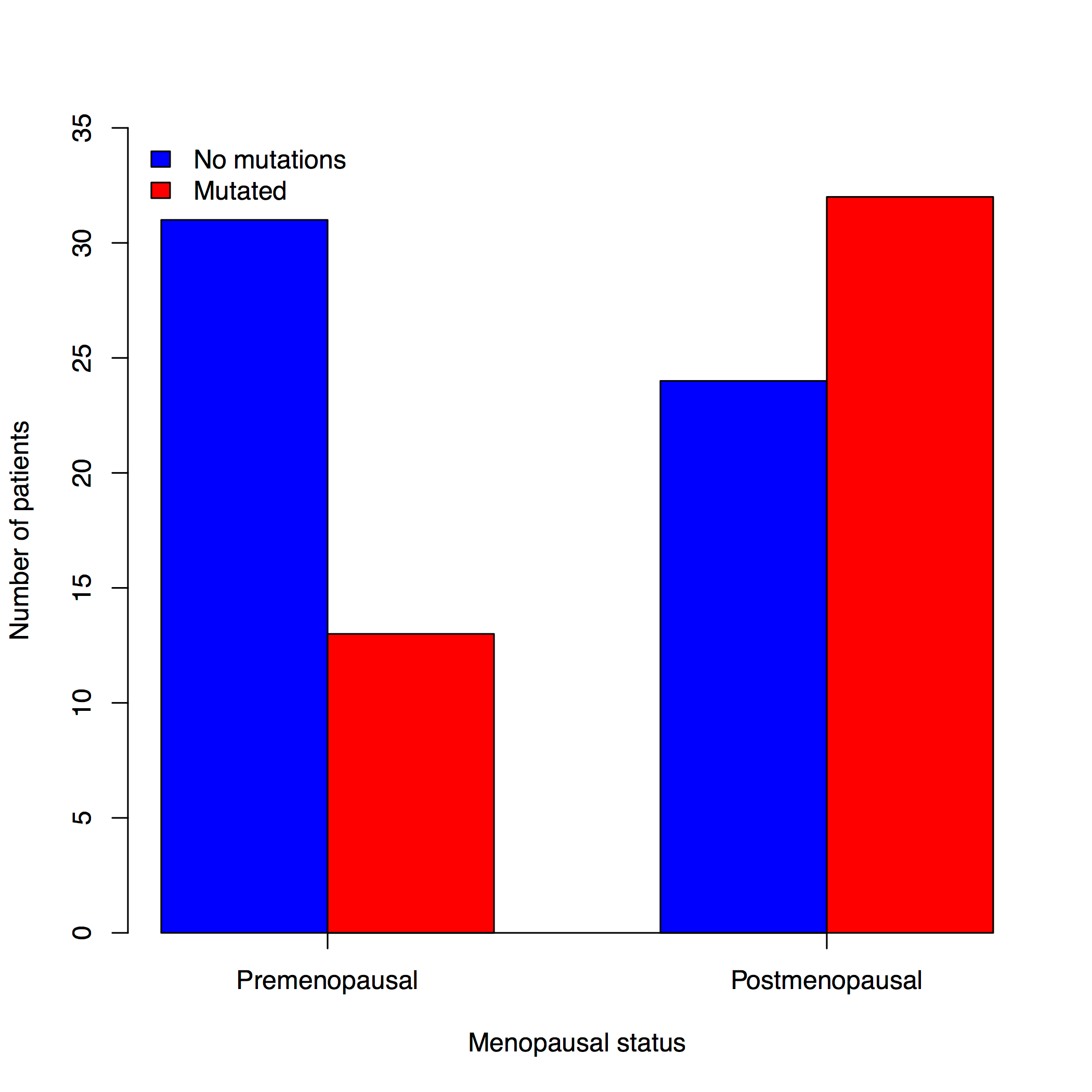

Supplement: S9 Fig — (TIFF) [file pmed.1002206.s009.tiff]

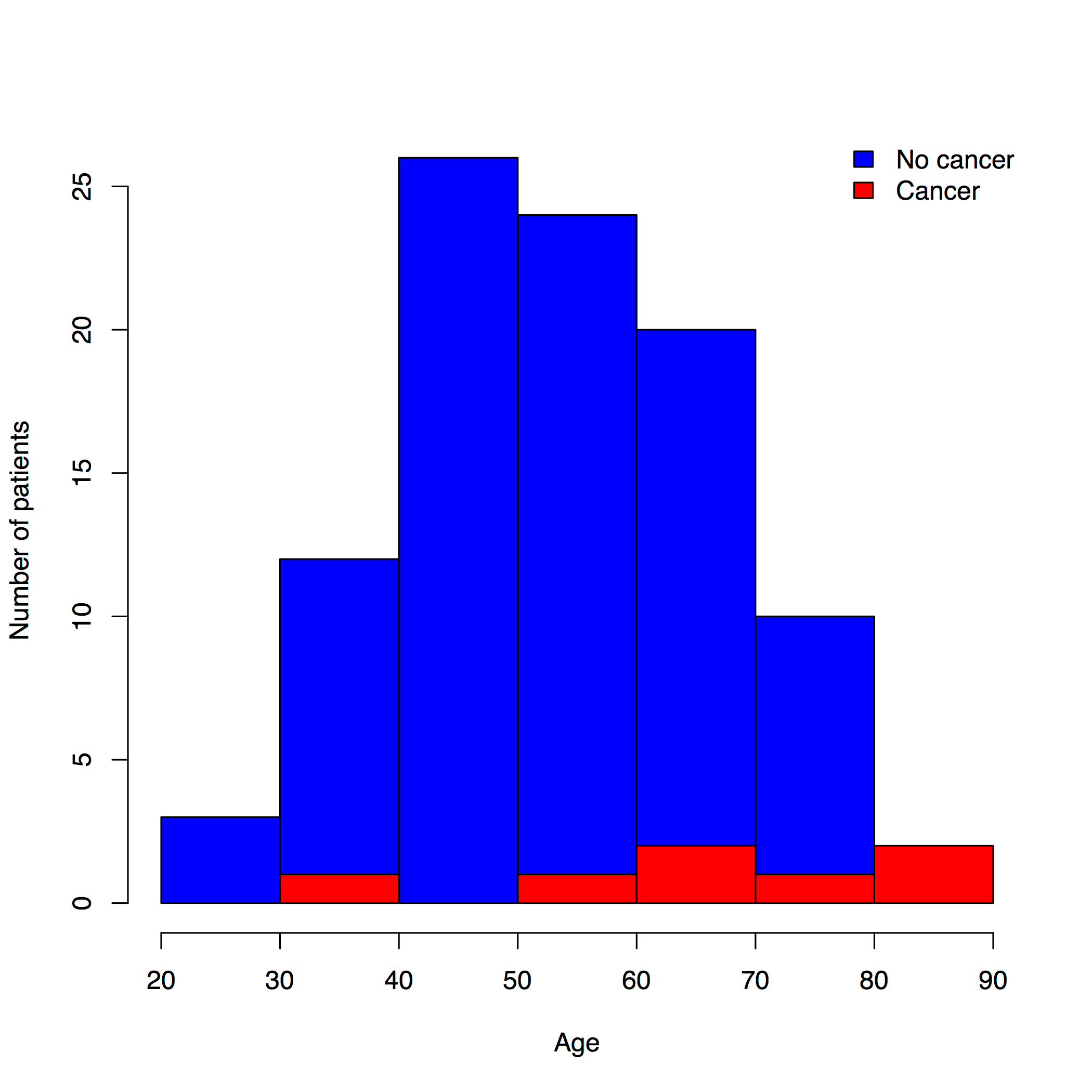

Supplement: S10 Fig — The mean age “No cancer” is equal to 53.23 y, the mean age for “Cancer” group is equal to 65.57 y; difference = 12.34, CI95% = [-0.54–∞]. (TIFF) [file pmed.1002206.s010.tiff]

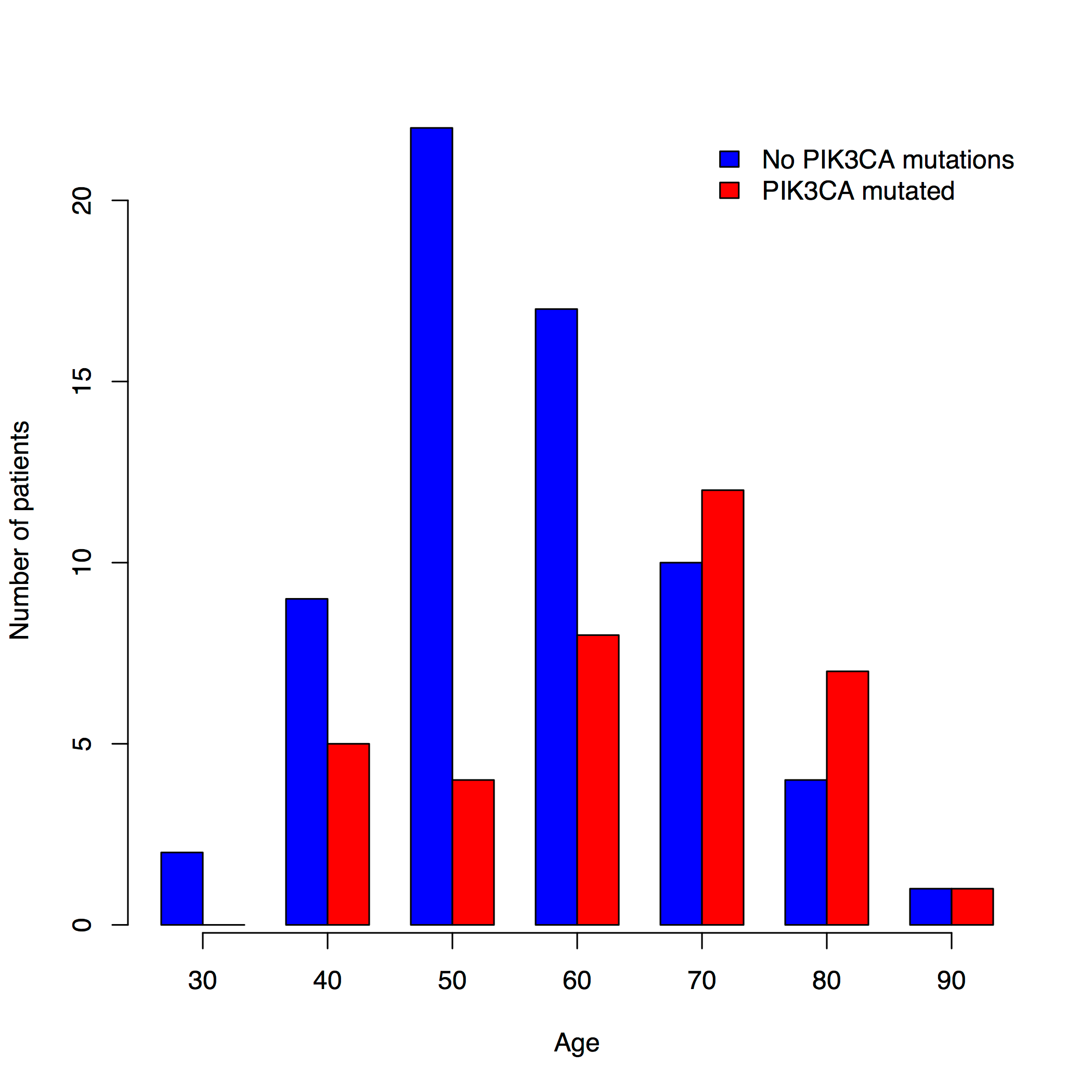

Supplement: S11 Fig — (TIFF) [file pmed.1002206.s011.tiff]

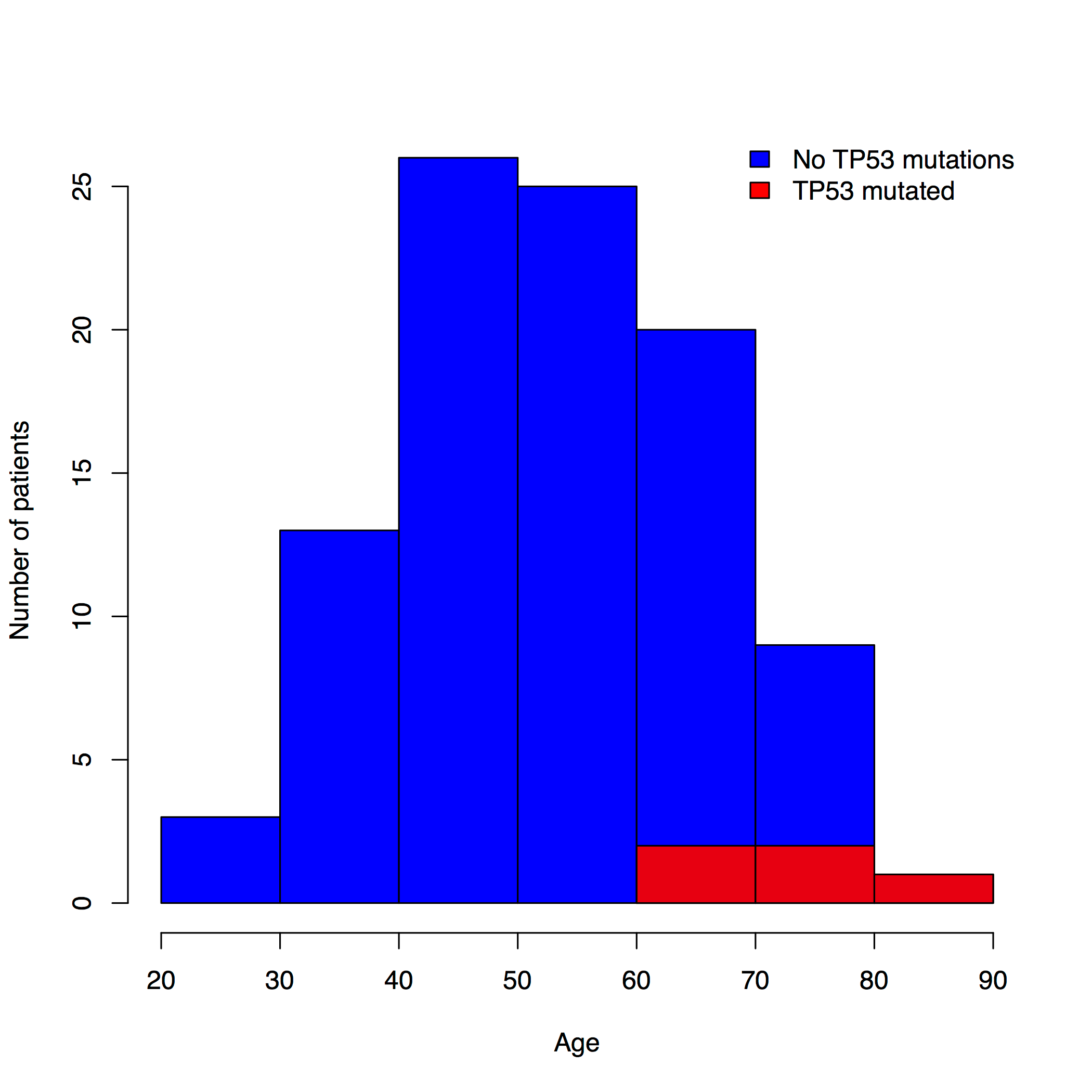

Supplement: S12 Fig — (TIFF) [file pmed.1002206.s012.tiff]
